# Postoperative outcomes in oesophagectomy with trainee involvement

**DOI:** 10.1093/bjsopen/zrab132

**Published:** 2022-01-17

**Authors:** Oesophago-Gastric Anastomosis Study Group (OGAA) on behalf of the West Midlands Research Collaborative, R P T Evans, S K Kamarajah, J Bundred, D Nepogodiev, J Hodson, R van Hillegersberg, J Gossage, R Vohra, E A Griffiths, P Singh, R P T Evans, J Hodson, S K Kamarajah, E A Griffiths, P Singh, D Alderson, J Bundred, R P T Evans, J Gossage, E A Griffiths, B Jefferies, S K Kamarajah, S McKay, I Mohamed, D Nepogodiev, K Siaw- Acheampong, P Singh, R van Hillegersberg, R Vohra, K Wanigasooriya, T Whitehouse, A Gjata, J I Moreno, F R Takeda, B Kidane, R Guevara Castro, T Harustiak, A Bekele, A Kechagias, I Gockel, A Kennedy, A Da Roit, A Bagajevas, J S Azagra, H A Mahendran, L Mejía-Fernández, B P L Wijnhoven, J El Kafsi, R H Sayyed, M Sousa, A S Sampaio, I Negoi, R Blanco, B Wallner, P M Schneider, P K Hsu, A Isik, S Gananadha, V Wills, M Devadas, C Duong, M Talbot, M W Hii, R Jacobs, N A Andreollo, B Johnston, G Darling, A Isaza-Restrepo, G Rosero, F Arias-Amézquita, D Raptis, J Gaedcke, D Reim, J Izbicki, J H Egberts, S Dikinis, D W Kjaer, M H Larsen, M P Achiam, J Saarnio, D Theodorou, T Liakakos, D P Korkolis, W B Robb, C Collins, T Murphy, J Reynolds, V Tonini, M Migliore, L Bonavina, M Valmasoni, R Bardini, J Weindelmayer, M Terashima, R E White, E Alghunaim, M Elhadi, A M Leon-Takahashi, H Medina-Franco, P C Lau, K E Okonta, J Heisterkamp, C Rosman, R van Hillegersberg, G Beban, R Babor, A Gordon, J I Rossaak, K M I Pal, A U Qureshi, S A Naqi, A A Syed, J Barbosa, C S Vicente, J Leite, J Freire, R Casaca, R C T Costa, R R Scurtu, S S Mogoanta, C Bolca, S Constantinoiu, D Sekhniaidze, M Bjelović, J B Y So, G Gačevski, C Loureiro, M Pera, A Bianchi, M Moreno Gijón, J Martín Fernández, M S Trugeda Carrera, M Vallve-Bernal, M A Cítores Pascual, S Elmahi, J Hedberg, S Mönig, S Gutknecht, M Tez, A Guner, T B Tirnaksiz, E Colak, B Sevinç, A Hindmarsh, I Khan, D Khoo, R Byrom, J Gokhale, P Wilkerson, P Jain, D Chan, K Robertson, S Iftikhar, R Skipworth, M Forshaw, S Higgs, J Gossage, R Nijjar, Y K S Viswanath, P Turner, S Dexter, A Boddy, W H Allum, S Oglesby, E Cheong, D Beardsmore, R Vohra, N Maynard, R Berrisford, S Mercer, S Puig, R Melhado, C Kelty, T Underwood, K Dawas, W Lewis, A Al-Bahrani, G Bryce, M Thomas, A T Arndt, F Palazzo, R A Meguid, J Fergusson, E Beenen, C Mosse, J Salim, S Cheah, T Wright, M P Cerdeira, P McQuillan, M Richardson, H Liem, J Spillane, M Yacob, F Albadawi, T Thorpe, A Dingle, C Cabalag, K Loi, O M Fisher, S Ward, M Read, M Johnson, R Bassari, H Bui, I Cecconello, R A A Sallum, J R M da Rocha, L R Lopes, V Tercioti, J D S Coelho, J A P Ferrer, G Buduhan, L Tan, S Srinathan, P Shea, J Yeung, F Allison, P Carroll, F Vargas-Barato, F Gonzalez, J Ortega, L Nino-Torres, T C Beltrán-García, L Castilla, M Pineda, A Bastidas, J Gómez-Mayorga, N Cortés, C Cetares, S Caceres, S Duarte, A Pazdro, M Snajdauf, H Faltova, M Sevcikova, P B Mortensen, N Katballe, T Ingemann, B Morten, I Kruhlikava, A P Ainswort, N M Stilling, J Eckardt, J Holm, M Thorsteinsson, M Siemsen, B Brandt, B Nega, E Teferra, A Tizazu, J S Kauppila, V Koivukangas, S Meriläinen, R Gruetzmann, C Krautz, G Weber, H Golcher, G Emons, A Azizian, M Ebeling, S Niebisch, N Kreuser, G Albanese, J Hesse, L Volovnik, U Boecher, M Reeh, S Triantafyllou, D Schizas, A Michalinos, E Baili, M Mpoura, A Charalabopoulos, D K Manatakis, D Balalis, J Bolger, C Baban, A Mastrosimone, O McAnena, A Quinn, C B Ó Súilleabháin, M M Hennessy, I Ivanovski, H Khizer, N Ravi, N Donlon, M Cervellera, S Vaccari, S Bianchini, L Sartarelli, E Asti, D Bernardi, S Merigliano, L Provenzano, M Scarpa, L Saadeh, B Salmaso, G De Manzoni, S Giacopuzzi, R La Mendola, C A De Pasqual, Y Tsubosa, M Niihara, T Irino, R Makuuchi, K Ishii, M Mwachiro, A Fekadu, A Odera, E Mwachiro, D AlShehab, H A Ahmed, A O Shebani, A Elhadi, F A Elnagar, H F Elnagar, S T Makkai-Popa, L F Wong, T Yunrong, S Thanninalai, H C Aik, P W Soon, T J Huei, H N L Basave, R Cortés-González, S M Lagarde, J J B van Lanschot, C Cords, W A Jansen, I Martijnse, R Matthijsen, S Bouwense, B Klarenbeek, M Verstegen, F van Workum, J P Ruurda, A van der Veen, J W van den Berg, N Evenett, P Johnston, R Patel, A MacCormick, M Young, B Smith, C Ekwunife, A H Memon, K Shaikh, A Wajid, N Khalil, M Haris, Z U Mirza, S B A Qudus, M Z Sarwar, A Shehzadi, A Raza, M H Jhanzaib, J Farmanali, Z Zakir, O Shakeel, I Nasir, S Khattak, M Baig, M A Noor, H H Ahmed, A Naeem, A C Pinho, R da Silva, H Matos, T Braga, C Monteiro, P Ramos, F Cabral, M P Gomes, P C Martins, A M Correia, J F Videira, C Ciuce, R Drasovean, R Apostu, C Ciuce, S Paitici, A E Racu, C V Obleaga, M Beuran, B Stoica, C Ciubotaru, V Negoita, I Cordos, R D Birla, D Predescu, P A Hoara, R Tomsa, V Shneider, M Agasiev, I Ganjara, D Gunjić, M Veselinović, T Babič, T S Chin, A Shabbir, G Kim, A Crnjac, H Samo, I Díez del Val, S Leturio, I Díez del Val, S Leturio, J M Ramón, M Dal Cero, S Rifá, M Rico, A Pagan Pomar, J A Martinez Corcoles, J L Rodicio Miravalles, S A Pais, S A Turienzo, L S Alvarez, P V Campos, A G Rendo, S S García, E P G Santos, E T Martínez, M J Fernández Díaz, C Magadán Álvarez, V Concepción Martín, C Díaz López, A Rosat Rodrigo, L E Pérez Sánchez, M Bailón Cuadrado, C Tinoco Carrasco, E Choolani Bhojwani, D P Sánchez, M E Ahmed, T Dzhendov, F Lindberg, M Rutegård, M Sundbom, C Mickael, N Colucci, A Schnider, S Er, E Kurnaz, S Turkyilmaz, A Turkyilmaz, R Yildirim, B E Baki, N Akkapulu, O Karahan, N Damburaci, R Hardwick, P Safranek, V Sujendran, J Bennett, Z Afzal, M Shrotri, B Chan, K Exarchou, T Gilbert, T Amalesh, D Mukherjee, S Mukherjee, T H Wiggins, R Kennedy, S McCain, A Harris, G Dobson, N Davies, I Wilson, D Mayo, D Bennett, R Young, P Manby, N Blencowe, M Schiller, B Byrne, D Mitton, V Wong, A Elshaer, M Cowen, V Menon, L C Tan, E McLaughlin, R Koshy, C Sharp, H Brewer, N Das, M Cox, W Al Khyatt, D Worku, R Iqbal, L Walls, R McGregor, G Fullarton, A Macdonald, C MacKay, C Craig, S Dwerryhouse, S Hornby, S Jaunoo, M Wadley, C Baker, M Saad, M Kelly, A Davies, F Di Maggio, S McKay, P Mistry, R Singhal, O Tucker, S Kapoulas, S Powell-Brett, P Davis, G Bromley, L Watson, R Verma, J Ward, V Shetty, C Ball, K Pursnani, A Sarela, H Sue Ling, S Mehta, J Hayden, N To, T Palser, D Hunter, K Supramaniam, Z Butt, A Ahmed, S Kumar, A Chaudry, O Moussa, A Kordzadeh, B Lorenzi, M Wilson, P Patil, I Noaman, J Willem, G Bouras, R Evans, M Singh, H Warrilow, A Ahmad, N Tewari, F Yanni, J Couch, E Theophilidou, J J Reilly, P Singh, G van Boxel, K Akbari, D Zanotti, B Sgromo, G Sanders, T Wheatley, A Ariyarathenam, A Reece-Smith, L Humphreys, C Choh, N Carter, B Knight, P Pucher, A Athanasiou, I Mohamed, B Tan, M Abdulrahman, J Vickers, K Akhtar, R Chaparala, R Brown, M M A Alasmar, R Ackroyd, K Patel, A Tamhankar, A Wyman, R Walker, B Grace, N Abbassi, N Slim, L Ioannidi, G Blackshaw, T Havard, X Escofet, A Powell, A Owera, F Rashid, P Jambulingam, J Padickakudi, H Ben-Younes, K McCormack, I A Makey, M K Karush, C W Seder, M J Liptay, G Chmielewski, E L Rosato, A C Berger, R Zheng, E Okolo, A Singh, C D Scott, M J Weyant, J D Mitchell

## Abstract

**Background:**

The complexity of oesophageal surgery and the significant risk of morbidity necessitates that oesophagectomy is predominantly performed by a consultant surgeon, or a senior trainee under their supervision. The aim of this study was to determine the impact of trainee involvement in oesophagectomy on postoperative outcomes in an international multicentre setting.

**Methods:**

Data from the multicentre Oesophago-Gastric Anastomosis Study Group (OGAA) cohort study were analysed, which comprised prospectively collected data from patients undergoing oesophagectomy for oesophageal cancer between April 2018 and December 2018. Procedures were grouped by the level of trainee involvement, and univariable and multivariable analyses were performed to compare patient outcomes across groups.

**Results:**

Of 2232 oesophagectomies from 137 centres in 41 countries, trainees were involved in 29.1 per cent of them (*n* = 650), performing only the abdominal phase in 230, only the chest and/or neck phases in 130, and all phases in 315 procedures. For procedures with a chest anastomosis, those with trainee involvement had similar 90-day mortality, complication and reoperation rates to consultant-performed oesophagectomies (*P* = 0.451, *P* = 0.318, and *P* = 0.382, respectively), while anastomotic leak rates were significantly lower in the trainee groups (*P* = 0.030). Procedures with a neck anastomosis had equivalent complication, anastomotic leak, and reoperation rates (*P* = 0.150, *P* = 0.430, and *P* = 0.632, respectively) in trainee-involved *versus* consultant-performed oesophagectomies, with significantly lower 90-day mortality in the trainee groups (*P* = 0.005).

**Conclusion:**

Trainee involvement was not found to be associated with significantly inferior postoperative outcomes for selected patients undergoing oesophagectomy. The results support continued supervised trainee involvement in oesophageal cancer surgery.

## Teaser

The aim of this study was to determine the impact of trainee involvement in oesophagectomy on perioperative outcomes in the international multicentre Oesophago-Gastric Anastomosis Audit (OGAA). Analysis of 2232 oesophagectomies has shown that trainee involvement did not negatively impact perioperative outcomes.

## Introduction

Oesophagectomy is associated with significant postoperative morbidity and mortality, with over 60 per cent of patients experiencing a postoperative complication, and reported 90-day mortality rates of almost 5 per cent^1–3^. The complexity of oesophageal surgery and the significant risk of negative outcomes necessitates that oesophagectomy is predominantly performed by a consultant surgeon, or a senior trainee under direct supervision.

Current evidence on the impact of trainee involvement in oesophagectomy is predominantly limited to single-centre, small-volume retrospective series, and analyses of the American College of Surgeons’ National Surgical Quality Improvement Program (NSQIP) database. These studies have suggested that, within structured supervised training, trainee input does not negatively impact on outcomes^4–9^. However, despite these findings, concerns remain around involving trainees in oesophagectomy, as other evidence from a variety of complex procedures from different surgical specialties has suggested increased morbidity with trainee involvement. For example, trainee involvement in major lower limb amputation is associated with increased major morbidity, increased operative time, and an increased need for intraoperative transfusions^10^. Similar findings have also been reported for appendicectomy, cholecystectomy, and bariatric procedures, with studies evaluating combined trainee-performed and trainee-supervised procedures suggesting that trainee involvement increased morbidity, operative time, and length of hospital stay^11^^,^^12^. In light of these findings, greater evaluation of the impact of trainee involvement in oesophagectomy is required, to determine the effect of training on patient outcomes.

Some countries publish surgeon-specific outcome data that are freely available to the public^13^^,^^14^. Although this leads to greater accountability, and the ability to compare outcomes across units, it could potentially create an environment where training opportunities are diminished due to fears that this could negatively impact on published outcome data^15^^,^^16^. As such, it is important to identify whether trainee involvement in oesophagectomy impacts patient outcome to dispel these fears, and ensure that the next generation of surgeons receives adequate training opportunities, in order to provide continued high-quality oesophageal surgery in the future. The Oesophago-Gastric Anastomosis Audit (OGAA) was an international multicentre cohort study, investigating perioperative outcomes for patients undergoing oesophagectomy for oesophageal cancer^1^^,^^17^^,^^18^. The aim of this present study was to use the data from the OGAA cohort to determine the impact of trainee involvement on postoperative outcomes after oesophagectomy in an international multicentre setting.

## Methods

### Study design of OGAA

The OGAA study was run by the Oesophago-Gastric Anastomosis Study Group, on behalf of the West Midlands Research Collaborative. The protocol for this study has previously been published^18^. Centres performing oesophagectomy for oesophageal cancer were invited to contribute to the OGAA cohort, and teams of surgeons, surgical trainees, research nurses, or medical students prospectively identified eligible patients over a 9-month period from 2 April 2018 to 31 December 2018. Patients were then followed up for 90 days after the date of oesophagectomy, to allow outcome data to be collected. At each centre, the nominated lead (consultant/attending only; see *Appendix S1*) was assigned overall responsibility for centre level data, for performing data validation, and for ensuring complete case ascertainment. External review was not performed.

### Surgeon designation and involvement

Operation-specific characteristics were recorded for each procedure, including details of the operative techniques used, and the designation of the primary surgeon performing each phase of the operation. The OGAA included centres from 41 countries, which used a variety of nomenclature for surgeon designation. As such, a ‘consultant surgeon’ was defined as a surgeon with an independent surgical practice inclusive of oesophagectomy. All surgeons that did not meet these criteria were defined as a ‘trainee’. The primary operating surgeon was recorded for each phase of the oesophagectomy, namely the abdomen and chest, as well as the neck phase (in procedures with neck anastomosis). Oesophagectomies were defined as ‘T_abdomen_’ where the trainee performed only the abdominal phase, ‘T_chest_’ where the trainee performed only the chest (and/or neck) phase, or as ‘T_abdomen+chest_’ where the trainee performed both the abdominal and chest (and/or neck) phase. Procedures with no trainee involvement were denoted as ‘T_neither_’.

### Outcome measures

The primary aim of the study was to assess the impact of trainee involvement on postoperative mortality, as defined as death within 90 days from surgery. Secondary outcomes included the rates and grades of either anastomotic leak or conduit necrosis, complication rates, length of stay, need for reoperation, and 30-day mortality. Complications were defined by the Esophageal Complications Consensus Group (ECCG) framework^19^, and were classified based on the Clavien–Dindo grade; the overall complication and major (grade III–V) complication rates were analysed as separate outcomes. All outcomes were analysed separately by the anastomosis location, as both operative difficulty and patient outcomes are known to differ between procedures with chest and neck anastomoses^1^^,^^20^.

Tumour staging was performed in accordance with the TNM eighth edition^21^. Positive longitudinal and circumferential tumour margins in the OGAA were defined as tumour identifiable 1 mm or less, in accordance with the Royal College of Pathologists guidance^22^.

### Ethical approval and data sharing for OGAA

Ethical approval was dependent on local protocols and was country specific. It was the responsibility of the local principal investigator of the enrolled unit to ensure appropriate ethical or audit approval was gained prior to commencement of the study. In the UK, the study was registered at each site as either a clinical audit or service evaluation, as it was an observational study designed to collect routine, anonymized data, with no change to the clinical care pathway.

### Statistical methods

Initially, cohort characteristics and outcomes were compared across the four groups of trainee involvement. Continuous variables were analysed using Kruskal–Wallis tests, and reported as mean (s.d.) if approximately normally distributed, with median and interquartile range (i.q.r.) used otherwise. Ordinal variables were also assessed using Kruskal–Wallis tests, with χ^2^ tests used for nominal variables.

For the primary outcomes, comparisons across the groups were then repeated using a generalized estimating equation approach, in order to account for potential non-independence of outcomes for patients treated at the same centre. As such, the centre was set as the subject variable, and the patient ID was the within-subject variable, with an exchangeable correlation structure used. All outcomes considered in this analysis were dichotomous; hence, a binary logistic model was used. Initially, univariable models were produced for each outcome, with the trainee involvement being the only independent variable. Multivariable models were then produced, to adjust for other potentially confounding factors. These used a backwards stepwise approach (removal at *P* > 0.1) to select other patient-, tumour-, and treatment-related factors for inclusion in the model. The goodness-of-fit of continuous variables was assessed graphically prior to producing the final model, with variables being divided into categories and treated as nominal where poor fit was detected. Where non-convergence of the model occurred owing to small within-group sample sizes, the offending variables were identified, and had categories combined to increase within-group sample sizes, where possible. Where this could not be meaningfully performed, patients from the affected category were excluded. The performance of the final multivariable models was quantified using the area under the receiver operating characteristic curve (AUROC), and Hosmer–Lemeshow tests.

All analyses were performed using SPSS 22 (IBM, Armonk, NY, USA), with *P* < 0.05 deemed to be indicative of statistical significance throughout.

## Results

### Cohort characteristics

Data were available for a total of 2247 oesophagectomies from 137 centres, of which 106, 30, and one were from high-, medium-, and low-income countries, respectively. Contributing centres had a median of three (i.q.r. 2–4) surgeons, 700 (i.q.r. 350–1020) total hospital beds, and 24 (i.q.r. 14–36) ICU beds. Seventy-one per cent of centres had a 24-hour on-call oesophageal surgery specialist, and 68.7 per cent had a 24-hour on-call interventional radiology specialist. Of the procedures recorded, 15 were excluded, either because no anastomosis was performed (four procedures), no anastomosis site was recorded (five procedures), or details of trainee involvement were not recorded (six procedures). As such, a total of 2232 procedures were included in the final analysis. Of these, 650 (29.1 per cent) had trainee involvement, with a trainee performing only the ‘abdominal’ phase in 230 procedures (T_abdomen_, 10.3 per cent), only the ‘chest and/or neck’ phase in 130 procedures (T_chest_, 4.7 per cent), and ‘both’ phases in 315 procedures (T_abdomen+chest_, 14.1 per cent).

**Fig. 1:**
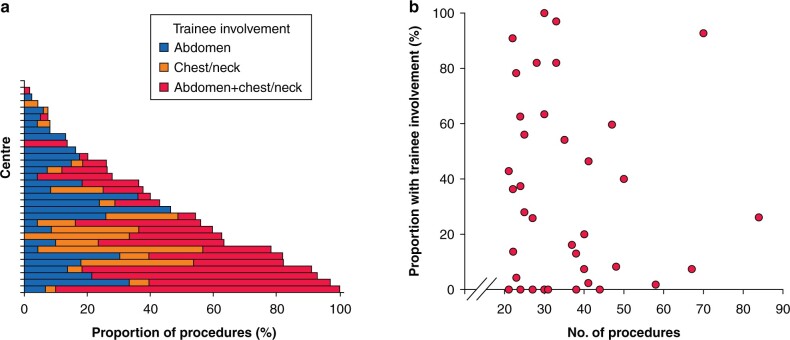
Proportion of procedures with trainee involvement **a** Per centre. **b** Per centre volume. Only the 41 centres that contributed more than 20 procedures to the analysis are included, such that percentages could be reliably estimated. All procedures were included, regardless of the location of the anastomosis.

The proportion of procedures with trainee involvement was found to differ significantly across centres (*P* < 0.001, *Fig. 1*). For the 41 centres that contributed more than 20 procedures to the analysis, the proportion of trainee-involved procedures ranged from 0 per cent (in 10 centres) to 100 per cent (in one centre). There was no evidence of a significant correlation between the centre volume and the proportion of trainee-involved oesophagectomies (Spearman’s rho: −0.046 (*P* = 0.775), *Fig. 1b*). However, trainee involvement rates were found to differ significantly by continent (*P* < 0.001; *Fig.S1*), with the lowest rates in Africa (17.7 per cent) and Europe (23.8 per cent), and the highest rates in Asia (62.5 per cent) and North America (75.2 per cent).

For subsequent analysis, procedures were divided by the site of the anastomosis, with 1722 (77.2 per cent) being located in the chest and 510 (22.8 per cent) in the neck.

### Chest anastomosis

#### Baseline characteristics

In procedures with an anastomosis in the chest, a trainee performed only the abdominal phase in 175 procedures (T_abdomen_, 10.2 per cent), only the chest phase in 93 procedures (T_chest_, 5.4 per cent), both phases in 198 procedures (T_abdomen+chest_, 11.5 per cent), and in neither phase in 1256 (T_neither_, 72.9 per cent). No significant differences in the distributions of age, sex, BMI, or ASA grade were detected between these four groups (*Table 1*). However, significant differences in rates of cardiovascular disease, current smokers, and squamous cell carcinoma histology were observed, all of which were more frequent in the T_neither_ group. In addition, a significant difference in Eastern Cooporative Oncology Group (ECOG) status was observed, being lower in the T_chest_ group, while the Charlson Comorbidity Index (CCI) was significantly lower in the T_abdomen+chest_ group.

**Table 1:** Cohort characteristics of procedures with chest anastomoses by trainee involvement

		Trainee involvement				
	*n*	Neither	Abdomen	Chest	Abdomen + chest	*P*-value
**Age (years)**	1722	64.8 (9.9)	65.2 (9.5)	66.0 (9.1)	63.2 (10.6)	0.101
**Sex (% male)**	1722	1029 (81.9)	140 (80.0)	75 (80.6)	155 (78.3)	0.632
**BMI (kg/m^2^)**	1717	26.7 (5.2)	27.2 (5.5)	26.6 (4.4)	26.9 (5.3)	0.725
**ASA grade**	1722					0.580*
1		164 (13.1)	15 (8.6)	15 (16.1)	30 (15.2)	
2		695 (55.3)	100 (57.1)	48 (51.6)	99 (50.0)	
3		386 (30.7)	59 (33.7)	30 (32.3)	62 (31.3)	
4		11 (0.9)	1 (0.6)	0 (0.0)	7 (3.5)	
**ECOG performance status**	1719					0.006*
0		783 (62.4)	115 (66.1)	73 (78.5)	135 (68.2)	
1		388 (30.9)	55 (31.6)	17 (18.3)	54 (27.3)	
2		71 (5.7)	3 (1.7)	3 (3.2)	7 (3.5)	
3		10 (0.8)	1 (0.6)	0 (0.0)	1 (0.5)	
4		2 (0.2)	0 (0.0)	0 (0.0)	1 (0.5)	
**Charlson Comorbidity Index**	1722	6 (5–6)	6 (5–6)	6 (5–6)	5 (4–6)	**0.048**
**COPD**	1722	152 (12.1)	24 (13.7)	5 (5.4)	26 (13.1)	0.204
**Diabetes**	1722	165 (13.1)	22 (12.6)	10 (10.8)	18 (9.1)	0.418
**Cardiovascular disease**	1722	208 (16.6)	18 (10.3)	11 (11.8)	21 (10.6)	**0.027**
**Smoking status**	1673					**0.027**
Never		448 (36.9)	71 (41.3)	31 (33.7)	85 (43.8)	
Ex-smoker		559 (46.0)	86 (50.0)	50 (54.3)	85 (43.8)	
Current		208 (17.1)	15 (8.7)	11 (12.0)	24 (12.4)	
**Histology**	1721					**0.020**
Adenocarcinoma		1024 (81.5)	158 (90.3)	79 (84.9)	172 (87.3)	
Squamous cell carcinoma		195 (15.5)	14 (8.0)	10 (10.8)	24 (12.2)	
Other		37 (2.9)	3 (1.7)	4 (4.3)	1 (0.5)	
**Tumour location**	1721					0.453
Distal/Siewert 1–2		1121 (89.3)	166 (94.9)	85 (91.4)	179 (90.9)	
Middle		91 (7.2)	7 (4.0)	4 (4.3)	11 (5.6)	
Proximal		5 (0.4)	0 (0.0)	0 (0.0)	0 (0.0)	
Siewert 3		39 (3.1)	2 (1.1)	4 (4.3)	7 (3.6)	
**TNM stage (on pathology)**	1706					0.469*
Stage 0		155 (12.5)	24 (13.8)	13 (14.0)	19 (9.6)	
Stage I		196 (15.8)	34 (19.5)	11 (11.8)	32 (16.2)	
Stage II		188 (15.1)	16 (9.2)	13 (14.0)	21 (10.7)	
Stage III		430 (34.6)	60 (34.5)	33 (35.5)	77 (39.1)	
Stage IV		273 (22.0)	40 (23.0)	23 (24.7)	48 (24.4)	

Continuous data are reported as median (i.q.r.) or mean (s.d.), with *P*-values obtained from Kruskal–Wallis tests. Categorical data are reported as *n* (column %), with *P*-values from χ^2^ tests, unless stated otherwise. Bold *P*-values are significant at < 0.05.

*P-value from Kruskal–Wallis test, as the factor is ordinal. ECOG, Eastern Cooperative Oncology Group; COPD, chronic obstructive pulmonary disease.

Comparison of the approach to treatment and surgery across the groups found several significant differences, including the use of pre- and postoperative nutritional support, neoadjuvant therapy, anastomotic technique, operative approach, and gastric tube size (*Table 2*).

**Table 2:** Treatment approach in procedures with chest anastomoses by trainee involvement

		Trainee involvement				
	*n*	Neither	Abdomen	Chest	Abdomen + chest	*P*-value
**Preoperative nutrition**	1720					**<** **0.001**
None		643 (51.3)	88 (50.3)	38 (40.9)	123 (62.1)	
Oral supplements		490 (39.1)	64 (36.6)	44 (47.3)	49 (24.7)	
Enteral tube nutrition		95 (7.6)	21 (12.0)	10 (10.8)	25 (12.6)	
Parenteral nutrition		26 (2.1)	2 (1.1)	1 (1.1)	1 (0.5)	
**Neoadjuvant therapy**	1722					**<** **0.001**
None		350 (27.9)	35 (20.0)	15 (16.1)	28 (14.1)	
Chemoradiotherapy		374 (29.8)	54 (30.9)	20 (21.5)	56 (28.3)	
Chemotherapy alone		530 (42.2)	86 (49.1)	58 (62.4)	114 (57.6)	
Radiotherapy alone		2 (0.2)	0 (0.0)	0 (0.0)	0 (0.0)	
**Postoperative nutrition**	1721					**<** **0.001**
None		539 (42.9)	60 (34.3)	35 (37.6)	90 (45.5)	
Feeding jejunostomy		591 (47.1)	108 (61.7)	53 (57.0)	104 (52.5)	
Nasojejunal tube		125 (10.0)	7 (4.0)	5 (5.4)	4 (2.0)	
**Anastomosis technique**	1722					**0.002**
Circular stapled		851 (67.8)	93 (53.1)	56 (60.2)	145 (73.2)	
Handsewn		175 (13.9)	34 (19.4)	16 (17.2)	19 (9.6)	
Linear stapled		230 (18.3)	48 (27.4)	21 (22.6)	34 (17.2)	
**Abdominal phase**	1711					**<** **0.001**
Minimally invasive		686 (55.1)	113 (64.6)	67 (72.0)	69 (34.8)	
Open		559 (44.9)	62 (35.4)	26 (28.0)	129 (65.2)	
**Thoracic phase**	1721					**<** **0.001**
Minimally invasive		323 (25.7)	88 (50.3)	4 (4.3)	45 (22.7)	
Open		932 (74.3)	87 (49.7)	89 (95.7)	153 (77.3)	
**Gastric tube**	1721					**<** **0.001**
Thin (< 5 cm)		719 (57.3)	121 (69.1)	40 (43.0)	114 (57.6)	
Wide/whole stomach		536 (42.7)	54 (30.9)	53 (57.0)	84 (42.4)	
**Pyloric procedures**	1722					0.200
Not performed		815 (64.9)	103 (58.9)	65 (69.9)	129 (65.2)	
Botox/dilatation		145 (11.5)	27 (15.4)	3 (3.2)	20 (10.1)	
Pyloromyotomy		31 (2.5)	6 (3.4)	1 (1.1)	4 (2.0)	
Pyloroplasty		265 (21.1)	39 (22.3)	24 (25.8)	45 (22.7)	
**Lymph nodes removed**	1714	24 (17–33)	24 (19–33)	23 (17–30)	23 (15–32)	0.261

Continuous data are reported as median (i.q.r.), with *P*-values obtained from Kruskal–Wallis tests. Categorical data are reported as *n* (column %), with *P*-values obtained from χ^2^ tests. Bold *P*-values are significant at < 0.05.

#### Postoperative outcomes

On univariable analysis, 90-day mortality rates were found to be similar across the four groups (*P* = 0.451; *Table 3*). Secondary outcomes, including 30-day mortality (*P* = 0.587), major complication rates (*P* = 0.933), and the proportion of cases requiring return to theatre (*P* = 0.382), were also not found to differ significantly between groups. However, a significant difference in the composite rate of anastomotic leak or conduit necrosis was observed, which was higher in procedures without trainee involvement than those where trainees completed at least one phase (14.0 per cent *versus* 6.3–11.6 per cent; *P* = 0.030). Duration of surgery also differed significantly between groups (*P* < 0.001), being longer when the trainee was involved in any phase of the procedure. The overall length of stay also differed significantly between groups (*P* = 0.010), tending to be shorter in procedures with trainee involvement (median 11 *versus* 12 days), while ICU stay tended to be longer in the T_chest_ and T_abdomen+chest_ groups (median 4 days *versus* 3 days in other groups; *P* = 0.009). The total number of lymph nodes removed and the rate of positive margins was not significantly different between groups (*P* = 0.261 and *P* = 0.129, respectively).

**Table 3:** Outcomes of procedures with chest anastomoses by trainee involvement

		Trainee involvement				
	*n*	Neither	Abdomen	Chest	Abdomen + chest	*P*-value
**90-day mortality**	1722	41 (3.3)	7 (4.0)	1 (1.1)	4 (2.0)	0.451
**Anastomotic leak/necrosis**	1722	176 (14.0)	11 (6.3)	10 (10.8)	23 (11.6)	**0.030**
**Anastomotic leak/necrosis grade**	1722					**0.035** *
None		1080 (86.0)	164 (93.7)	83 (89.2)	175 (88.4)	
Grade 1		75 (6.0)	4 (2.3)	4 (4.3)	10 (5.1)	
Grade 2		60 (4.8)	4 (2.3)	2 (2.2)	3 (1.5)	
Grade 3		41 (3.3)	3 (1.7)	4 (4.3)	10 (5.1)	
**Any complication**	1722	763 (60.7)	109 (62.3)	62 (66.7)	132 (66.7)	0.318
**Clavien–Dindo Grade III–V complication**	1722	307 (24.4)	43 (24.6)	20 (21.5)	49 (24.7)	0.933
**Duration of surgery (min)**	1709	341 (270–420)	380 (315–450)	380 (330–420)	371 (300–420)	**<** **0.001**
**Positive margins**	1722	243 (19.3)	29 (16.6)	26 (28.0)	43 (21.7)	0.129
**ICU length of stay (days)**	1714	3 (1–7)	3 (1–5)	4 (2–6)	4 (2–7)	**0.010**
**Total length of stay (days)**	1714	12 (9–18)	11 (9–15)	11 (9–17)	11 (9–15)	**0.009**
**Return to theatre**	1722	144 (11.5)	18 (10.3)	7 (7.5)	28 (14.1)	0.382
**30-day mortality**	1722	25 (2.0)	3 (1.7)	0 (0.0)	4 (2.0)	0.587

Continuous data are reported as median (i.q.r.), with *P*-values obtained from Kruskal–Wallis tests. Categorical data are reported as *n* (column %), with *P*-values obtained from χ^2^ tests. Bold *P*-values are significant at < 0.05.

*P-value from Kruskal–Wallis test, as the factor is ordinal.

Rates of 90-day mortality, anastomotic leaks or conduit necrosis, and Clavien–Dindo Grade III–V complications were then assessed using multivariable analysis (*Table 4*; *Tables S1* and *S2*). It was not possible to produce a reliable multivariable analysis of 90-day mortality, in light of the low event rate. For the other outcomes assessed, the multivariable models had reasonable performance, with AUROCs of 0.60–0.65, and *P* > 0.05 on Hosmer–Lemeshow tests. Overall rates of anastomotic leaks or conduit necrosis were significantly reduced in trainee-involved procedures on multivariable analysis (*P* = 0.043), with the adjusted rates being lowest in the T_abdomen_ group (*versus* T_neither_; odds ratio (OR) 0.45, 95 per cent c.i. 0.25–0.80; *P* = 0.006).

**Table 4:** Summary of univariable and multivariable models of primary outcomes in chest anastomoses

	90-day mortality		Anastomotic leak/conduit necrosis		Clavien-Dindo grade III–V complication	
	OR (95% c.i.)	*P*-value	OR (95% c.i.)	*P*-value	OR (95% c.i.)	*P*-value
**Univariable models**						
**Trainee involvement**		0.258		**0.012**		0.963
Neither	–	–	–	–	–	–
Abdomen	1.23 (0.63–2.43)	0.543	0.42 (0.25–0.71)	**0.001**	1.06 (0.72–1.57)	0.775
Chest	0.32 (0.05–1.93)	0.213	0.76 (0.44–1.32)	0.331	0.90 (0.51–1.58)	0.704
Abdomen + chest	0.60 (0.25–1.44)	0.252	0.79 (0.49–1.30)	0.356	0.99 (0.60–1.63)	0.957
**Summary of multivariable models**						
**Trainee involvement**		NA*		**0.043**		0.896
Neither	–	–	–	–	–	–
Abdomen	–	–	0.45 (0.25–0.80)	**0.006**	1.11 (0.73–1.70)	0.625
Chest	–	–	0.73 (0.40–1.32)	0.293	0.88 (0.48–1.60)	0.668
Abdomen + chest	–	–	0.94 (0.60–1.48)	0.788	1.03 (0.63–1.67)	0.919

Univariable analyses are from generalized estimating equation models, accounting for correlations between procedures from the same centre. Multivariable analyses extend these models to additionally adjust for all factors in *Tables 1* and *2***—**full details of the multivariable models are reported in *Tables S1* and *S2*. Bold *P*-values are significant at < 0.05. OR, odds ratio; c.i., confidence interval.

*It was not possible to produce a multivariable model of 90 day mortality, due to the low event rate.

### Neck anastomosis

#### Baseline characteristics

The analysis was then repeated for the subgroup of 510 procedures with anastomoses in the neck. Of these, a trainee performed only the abdominal phase in 55 procedures (T_abdomen_, 10.8 per cent), the chest and/or neck phase only in 12 procedures (T_chest_, 2.4 per cent), both phases in 117 procedures (T_abdomen+chest_, 22.9 per cent), and in neither phase in 326 procedures (T_neither_, 63.9 per cent). Comparison of cohort characteristics across these groups found significant differences in age, ECOG status, CCI, smoking status, and tumour location (*Table 5*). In addition, differences in a range of factors relating to the operative approach were observed (*Table 6*).

**Table 5:** Cohort characteristics of procedures with neck anastomoses by trainee involvement

		Trainee involvement				
	*n*	Neither	Abdomen	Chest/neck	Abdomen + chest/neck	*P*-value
**Age (years)**	510	63.4 ± 11.6	58.3 ± 12.1	64.3 ± 8.7	56.6 ± 12.2	**<** **0.001**
**Sex (% male)**	510	236 (72.4)	35 (63.6)	9 (75.0)	77 (65.8)	0.381
**BMI (kg/m^2^)**	510	24.6 ± 5.0	23.7 ± 4.1	25.0 ± 5.0	23.6 ± 5.2	0.135
**ASA grade**	510					0.072*
1		45 (13.8)	8 (14.5)	3 (25.0)	13 (11.1)	
2		188 (57.7)	36 (65.5)	1 (8.3)	86 (73.5)	
3		91 (27.9)	10 (18.2)	8 (66.7)	18 (15.4)	
4		2 (0.6)	1 (1.8)	0 (0.0)	0 (0.0)	
**ECOG performance status**	508					**0.043** *
0		154 (47.2)	23 (42.6)	7 (58.3)	69 (59.5)	
1		139 (42.6)	28 (51.9)	5 (41.7)	43 (37.1)	
2		30 (9.2)	3 (5.6)	0 (0.0)	4 (3.4)	
3		3 (0.9)	0 (0.0)	0 (0.0)	0 (0.0)	
4		0 (0.0)	0 (0.0)	0 (0.0)	0 (0.0)	
**Charlson Comorbidity Index**	510	6 (5–7)	5 (4–6)	6 (5–7)	5 (4–6)	**<** **0.001**
**COPD**	510	55 (16.9)	9 (16.4)	4 (33.3)	31 (26.5)	0.075
**Diabetes**	510	43 (13.2)	5 (9.1)	1 (8.3)	6 (5.1)	0.107
**Cardiovascular disease**	510	59 (18.1)	11 (20.0)	2 (16.7)	10 (8.5)	0.088
**Smoking status**	496					**0.019**
Never		110 (34.9)	29 (55.8)	3 (25.0)	57 (48.7)	
Ex-smoker		147 (46.7)	14 (26.9)	8 (66.7)	46 (39.3)	
Current		58 (18.4)	9 (17.3)	1 (8.3)	14 (12.0)	
**Histology**	510					0.249
Adenocarcinoma		146 (44.8)	25 (45.5)	7 (58.3)	37 (31.6)	
Squamous cell carcinoma		171 (52.5)	29 (52.7)	5 (41.7)	77 (65.8)	
Other		9 (2.8)	1 (1.8)	0 (0.0)	3 (2.6)	
**Tumour location**	510					**0.019**
Distal/Siewert 1–2		191 (58.6)	43 (78.2)	7 (58.3)	78 (66.7)	
Middle		84 (25.8)	7 (12.7)	4 (33.3)	31 (26.5)	
Proximal		44 (13.5)	4 (7.3)	0 (0.0)	4 (3.4)	
Siewert 3		7 (2.1)	1 (1.8)	1 (8.3)	4 (3.4)	
**TNM stage (on pathology)**	504					0.076*
Stage 0		60 (18.8)	17 (30.9)	2 (16.7)	34 (29.1)	
Stage I		44 (13.8)	5 (9.1)	2 (16.7)	17 (14.5)	
Stage II		70 (21.9)	11 (20.0)	3 (25.0)	25 (21.4)	
Stage III		91 (28.4)	13 (23.6)	3 (25.0)	28 (23.9)	
Stage IV		55 (17.2)	9 (16.4)	2 (16.7)	13 (11.1)	

Continuous data are reported as median (i.q.r.) or mean (s.d.), with *P*-values obtained from Kruskal–Wallis tests. Categorical data are reported as *n* (column %), with *P*-values from χ^2^ tests, unless stated otherwise. Bold *P*-values are significant at < 0.05. COPD, chronic obstructive pulmonary disease.

*P-value from Kruskal–Wallis test, as the factor is ordinal.

**Table 6:** Treatment approach in procedures with neck anastomoses by trainee involvement

		Trainee involvement				
	*n*	Neither	Abdomen	Chest/neck	Abdomen + chest/neck	*P*-value
**Preoperative nutrition**	510					**<** **0.001**
None		164 (50.3)	21 (38.2)	2 (16.7)	35 (29.9)	
Oral supplements		112 (34.4)	29 (52.7)	7 (58.3)	39 (33.3)	
Enteral tube nutrition		44 (13.5)	5 (9.1)	3 (25.0)	43 (36.8)	
Parenteral nutrition		6 (1.8)	0 (0.0)	0 (0.0)	0 (0.0)	
**Neoadjuvant therapy**	510					0.128
* *None		89 (27.3)	11 (20.0)	5 (41.7)	18 (15.4)	
* *Chemoradiotherapy		174 (53.4)	35 (63.6)	6 (50.0)	80 (68.4)	
Chemotherapy alone		59 (18.1)	9 (16.4)	1 (8.3)	19 (16.2)	
Radiotherapy alone		4 (1.2)	0 (0.0)	0 (0.0)	0 (0.0)	
**Postoperative nutrition**	510					**0.002**
None		86 (26.4)	10 (18.2)	4 (33.3)	28 (23.9)	
Feeding jejunostomy		166 (50.9)	31 (56.4)	5 (41.7)	39 (33.3)	
Nasojejunal tube		74 (22.7)	14 (25.5)	3 (25.0)	50 (42.7)	
**Anastomosis technique**	510					**<** **0.001**
Circular stapled		14 (4.3)	0 (0.0)	0 (0.0)	3 (2.6)	
Handsewn		200 (61.3)	46 (83.6)	10 (83.3)	100 (85.5)	
Linear stapled		112 (34.4)	9 (16.4)	2 (16.7)	14 (12.0)	
**Abdominal phase**	510					0.479
Minimally invasive		159 (48.8)	30 (54.5)	8 (66.7)	63 (53.8)	
Open		167 (51.2)	25 (45.5)	4 (33.3)	54 (46.2)	
**Thoracic phase**	506					**0.045**
Minimally invasive		165 (50.8)	33 (61.1)	5 (41.7)	74 (64.3)	
Open		160 (42.9)	21 (38.9)	7 (58.3)	41 (35.7)	
**Gastric tube**	509					**<** **0.001**
Thin (< 5 cm)		166 (51.1)	43 (78.2)	9 (75.0)	107 (91.5)	
Wide/whole stomach		159 (48.9)	12 (21.8)	3 (25.0)	10 (8.5)	
**Pyloric procedures**	510					**<** **0.001**
Not performed		233 (71.5)	23 (41.8)	11 (91.7)	34 (29.1)	
Botox/dilatation		16 (4.9)	14 (25.5)	0 (0.0)	26 (22.2)	
Pyloromyotomy		14 (4.3)	4 (7.3)	0 (0.0)	47 (40.2)	
Pyloroplasty		63 (19.3)	14 (25.5)	1 (8.3)	10 (8.5)	
**Lymph nodes removed**	509	19 (12–24)	18 (15–29)	16 (7–20)	18 (14–24)	0.220

Continuous data are reported as median (i.q.r.), with *P*-values obtained from Kruskal–Wallis tests. Categorical data are reported as *n* (column %), with *P*-values obtained from χ^2^ tests. Bold *P*-values are significant at < 0.05.

#### Postoperative outcomes

Univariable analysis found significant differences in both 90-day mortality rates (*P* = 0.005) and Clavien–Dindo Grade III–V complication rates (*P* = 0.028) between the four groups, both of which were lower in the groups with trainee involvement (*Table 7*). There was no significant difference in the rate of anastomotic leak/conduit necrosis (*P* = 0.430). Duration of surgery was not significantly different between groups (*P* = 0.133). The overall length of stay and ICU stay were significantly shorter in procedures with trainee involvement (*P* = 0.013 and *P* = 0.033, respectively). The number of lymph nodes removed did not significantly differ between groups (*P* = 0.220); however, procedures without trainee involvement were significantly more likely to have positive margins (*P* = 0.041).

**Table 7:** Outcomes of procedures with neck anastomoses by trainee involvement

		Trainee involvement				
	*n*	Neither	Abdomen	Chest/neck	Abdomen + chest/neck	*P*-value
**90-day mortality**	510	39 (12.0)	1 (1.8)	0 (0.0)	4 (3.4)	**0.005**
**Anastomotic leak/necrosis**	510	69 (21.2)	10 (18.2)	4 (33.3)	19 (16.2)	0.430
**Anastomotic leak/necrosis grade**	510					0.453*
None		257 (78.8)	45 (81.8)	8 (66.7)	98 (83.8)	
Grade 1		44 (13.5)	6 (10.9)	3 (25.0)	13 (11.1)	
Grade 2		9 (2.8)	0 (0.0)	0 (0.0)	1 (0.9)	
Grade 3		16 (4.9)	4 (7.3)	1 (8.3)	5 (4.3)	
**Any complication**	510	234 (71.8)	31 (56.4)	8 (66.7)	81 (69.2)	0.150
**Clavien–Dindo grade III–V complication**	510	108 (33.1)	14 (25.5)	3 (25.0)	22 (18.8)	**0.028**
**Duration of surgery (min)**	500	348 (250–480)	320 (255–364)	360 (318–450)	330 (274–380)	0.133
**Positive margins**	510	50 (15.3)	7 (12.7)	2 (16.7)	6 (5.1)	**0.041**
**ICU length of stay (days)**	505	4 (2–7)	2 (1–5)	2 (1–5)	3 (1–7)	**0.033**
**Total length of stay (days)**	505	14 (10–23)	12 (8–17)	21 (11–29)	12 (9–17)	**0.013**
**Return to theatre**	510	45 (13.8)	9 (16.4)	1 (8.3)	12 (10.3)	0.632
**30-day mortality**	510	31 (9.5)	1 (1.8)	0 (0.0)	4 (3.4)	**0.036**

Continuous data are reported as median (i.q.r.), with *P*-values obtained from Kruskal–Wallis tests. Categorical data are reported as *n* (column %), with *P*-values from χ^2^ tests, unless stated otherwise. Bold *P*-values are significant at < 0.05.

*P-value from Kruskal–Wallis test, as the factor is ordinal.

Multivariable analysis was not possible for the outcome of 90-day mortality, on account of the small number of events. After adjustment for other confounding factors, the difference between groups in the rate of Clavien–Dindo grade III–V complications became non-significant (*P* = 0.185; *Table 8* and *TablesS1* and *S2*).

**Table 8:** Summary of univariable and multivariable models of primary outcomes in neck anastomoses

	90-day mortality		Anastomotic leak/conduit necrosis		Clavien-Dindo grade III–V complication	
	OR (95% c.i.)	*P*-value	OR (95% c.i.)	*P*-value	OR (95% c.i.)	*P*-value
**Univariable models**						
**Trainee involvement**		**0.014**		0.380		**0.041**
Neither	–	–	–	–	–	–
Abdomen	NA*	NA*	1.13 (0.64–1.97)	0.680	0.63 (0.31–1.27)	0.199
Chest	NA*	NA*	1.59 (0.49–5.20)	0.442	0.73 (0.22–2.37)	0.595
Abdomen + chest	0.30 (0.11–0.78)*	**0.014** *	0.77 (0.48–1.26)	0.303	0.50 (0.31–0.80)	**0.004**
**Summary of multivariable models**						
**Trainee involvement**		NA*^,^†		0.090		0.185
Neither	–	–			–	–
Abdomen	–	–	0.90 (0.36–2.22)	0.820	0.58 (0.24–1.38)	0.217
Chest	–	–	1.46 (0.34–6.20)	0.611	0.69 (0.21–2.30)	0.541
Abdomen + chest	–	–	0.47 (0.24–0.90)	0.022	0.55 (0.31–0.99)	0.045

Univariable analyses are from generalised estimating equation models, accounting for correlations between procedures from the same centre. Multivariable analyses extend these models to additionally adjust for all factors in *Tables 1* and *2***—**full details of the multivariable models are reported in *Tables S1* and *S2*. Bold *P*-values are significant at < 0.05. OR, odds ratio;

*OR represents a comparison of any trainee involvement *versus* no trainee involvement, due to the within-group sample sizes being insufficient to produce a reliable model comparing across four groups. †It was not possible to produce a multivariable model of 90-day mortality, due to the low event rate.

## Discussion

This analysis of an international multicentre cohort found no evidence to suggest that trainee involvement in oesophagectomy negatively impacts on postoperative outcome. Postoperative mortality, anastomotic leak rate, and complications were not significantly inferior when a trainee performed all or part of an oesophagectomy. Importantly, some key postoperative outcome measures, including the anastomotic leak rate and length of stay in patients with an anastomosis in the chest, were found to be superior in procedures with trainee involvement. Patients undergoing oesophagectomy with trainee involvement were found to be significantly less comorbid and had different treatment approaches, as compared to consultant-performed oesophagectomy, suggesting that appropriate patient selection for training procedures occurred, which may have helped ensure safe patient outcomes.

Concerns exist about the safety of trainee involvement in complex surgery. For example, trainee-performed hepatectomy and pancreatectomy have been shown to be associated with increased complication rates and operative times^23^. In the case of oesophagectomy, Phillips *et al*. reported on outcomes of 323 open Ivor Lewis procedures, 75 per cent of which had a trainee performing at least one stage. They reported no significant difference in pre-operative co-morbidity or staging and, furthermore, no significant differences in postoperative outcomes or 2-year survival rates in trainee- *versus* consultant-performed oesophagectomy^5^. Handagala *et al*. reported on 323 oesophagectomies, which used a variety of techniques, with 37 per cent being performed by a trainee. They also found no significant differences in patient comorbidities or preoperative treatment, or in postoperative morbidity or mortality between procedures with or without trainee involvement. Finally, Baron *et al*. reported on a similar case mix, including 241 open thoracoabdominal two- and three-stage oesophagectomies, 35 per cent of which were performed by a trainee. However, they found trainee-performed oesophagectomies to have significantly higher anastomotic leak rates (consultant 7 per cent *versus* trainee 20 per cent), although this did not lead to a significant difference in postoperative mortality or survival between the two groups^4^.

Saliba *et al*., using NSQIP data, reported outcomes from nine different surgical specialties on 1 349 684 patients, appraising trainee and patient outcomes. Procedures with trainee involvement were performed on younger, more functionally independent patients with a lower BMI but higher ASA grades. Subsequent postoperative morbidity was comparable, operative duration was longer, and overall mortality was lower when trainees were involved^24^. A similar analysis of NSQIP data by Ferraris *et al*. in 266 411 procedures used propensity matching to control for baseline differences between groups, and showed that, although procedures with trainee involvement were associated with increased morbidity, mortality rates were comparable^25^. Patients with similar levels of complications were increasingly likely to suffer ‘failure to rescue’ in consultant-performed cases, demonstrating that trainee involvement may be protective for patients. In a NSQIP analysis by Cobb *et al*., propensity-matched patients undergoing oesophagectomy had a significantly lower mortality in trainee-performed cases^8^. Khoushhal *et al*. evaluated outcomes for 5142 oesophagectomies from the NSQIP database and found that neither surgical specialty (cardiothoracic, general surgery) nor trainee involvement influenced mortality^9^. However, a major limitation when evaluating trainee involvement in procedures using NSQIP data is that the NSQIP definition of trainee involvement is trainee ‘in’ or ‘not in’ the operating room, and does not define the degree to which the trainee is involved (assisting or performing), as is the case of the presented analysis of the OGAA cohort.

There remains a lack of data analysing the effect of training in minimally invasive oesophagectomy (MIE). The OGAA study provides postoperative outcomes on 2232 oesophagectomies; trainees were involved in 28.4 per cent of open *versus* 26.4 per cent of hybrid and 32.9 per cent of MIE procedures, demonstrating that modern trainees are receiving similar levels of exposure to both open and minimally invasive oesophageal surgery. The learning curve associated with MIE is associated with a significant increase in postoperative morbidity, including an associated increase in anastomotic leak rates of an additional 10 per cent^26^^,^^27^. In a retrospective study of 2121 consultant-performed Ivor Lewis MIEs, Claassen *et al*. showed that the length of the learning curve for textbook outcome was 46 cases, after which a plateau was reached, with 44.0 per cent achieving a textbook outcome^28^. Evidence from novel robotic oesophagectomy training programmes shows that safety can be maintained while reducing the learning curve to 22 cases^29^.

The current study has some limitations. There was considerable variability in rates of trainee involvement between centres, which varied from 0 to 100 per cent. This may have contributed to the significant differences between groups in the factors relating to the operative approach, which is generally centre related, and so may have introduced bias, particularly if higher-quality centres were more likely to engage in training. In an attempt to negate such confounding, multivariable models were used to adjust for within-centre correlation of outcomes, and for baseline differences between groups. However, these were limited by the small within-group sample sizes and the low event rates for some outcomes, hence residual confounding may remain. The small within-group sample sizes, particularly for the subgroup of neck anastomoses, will also have reduced the statistical power of the comparisons across the groups of trainee involvement. This will have increased the minimal detectable effect sizes, resulting in an increased false-negative rate. When defining the groups, surgeons were classified as either ‘consultant’ or ‘trainee’. However, training grade is known to convey differing levels of autonomy and ability, depending on the level of experience. Owing to the variability in nomenclature and training structures across countries, it was not possible to ascertain further details about the grade or level of experience of trainees; hence, it was not possible to assess variability in outcome within subgroups of trainees^30^. It was also not possible to identify cases where the primary surgeon was a consultant who had taken over from the trainee due to operative difficulty which, in such cases, may be at higher risk of negative outcomes, owing to increased operative difficulty.

## Collaborators

R.P.T. Evans, S.K. Kamarajah, J. Bundred, D. Nepogodiev, J. Hodson, R. van Hillegersberg, J. Gossage, R. Vohra, E.A. Griffiths, P. Singh, R.P.T. Evans, J. Hodson, S.K. Kamarajah, E.A. Griffiths, P. Singh, D. Alderson, J. Bundred, R.P.T. Evans, J. Gossage, E.A. Griffiths, B. Jefferies, S.K. Kamarajah, S. McKay, I. Mohamed, D. Nepogodiev, K. Siaw- Acheampong, P. Singh, R. van Hillegersberg, R. Vohra, K. Wanigasooriya, T. Whitehouse, A. Gjata (Albania), J.I. Moreno (Argentina), F.R. Takeda (Brazil), B. Kidane (Canada), R. Guevara Castro (Colombia), T. Harustiak (Czech Republic), A. Bekele (Ethiopia), A. Kechagias (Finland), I. Gockel (Germany), A. Kennedy (Ireland), A. Da Roit (Italy), A. Bagajevas (Lithuania), J.S. Azagra (Luxembourg), H.A. Mahendran (Malaysia), L. Mejía-Fernández (Mexico), B.P.L. Wijnhoven (The Netherlands), J. El Kafsi (New Zealand), R.H. Sayyed (Pakistan), M. Sousa (Portugal), A.S. Sampaio (Portugal), I. Negoi (Romania), R. Blanco (Spain), B. Wallner (Sweden), P.M. Schneider (Switzerland), P.K. Hsu (Taiwan), A. Isik (Turkey), S. Gananadha (The Canberra Hospital, Australia); V. Wills (John Hunter Hospital, Australia); M. Devadas (Nepean Hospital, Australia); C. Duong (Peter MacCallum Cancer Centre, Australia); M. Talbot (St George Public and Private Hospitals, Australia); M.W. Hii (St Vincent’s Hospital Melbourne, Australia); R. Jacobs (Western Hospital, Victoria, Australia); N.A. Andreollo (Unicamp University Hospital, Brazil); B. Johnston (Saint John Regional Hospital, Canada); G. Darling (Toronto General Hospital, University Health Network, Canada); A. Isaza-Restrepo (Hospital Universitario Mayor Mederi-Universidad del Rosario, Colombia); G. Rosero (Hospital San Ignacio-Universidad Javeriana, Colombia); F. Arias- Amézquita (University Hospital Fundacion Santafe de Bogota, Colombia); D. Raptis (University Clinic of Erlangen, Germany); J. Gaedcke (Medical Unversity Goettingen, Germany); D. Reim (Klinikum Rechts der Isar der TU München, Germany); J. Izbicki (University Hospital Hamburg Eppendorf, Germany); J.H. Egberts (University Hospital Kiel, Germany); S. Dikinis (Aalborg University Hospital, Denmark); D.W. Kjaer (Aarhus University Hospital, Denmark); M.H. Larsen (Odense University Hospital, Denmark); M.P. Achiam (Copenhagen University hospital Rigshospitalet, Denmark); J. Saarnio (Oulu University Hospital, Finland); D. Theodorou (Hippokration General Hospital University of Athens, Greece); T. Liakakos (Laikon General Hospital, Greece); D.P. Korkolis (St. Savvas Cancer Hospital, Greece); W.B. Robb (Beaumont Hospital, Ireland); C. Collins (University Hospital Galway, Ireland); T. Murphy (Mercy University Hospital, Ireland); J. Reynolds (St James's Hospital, Ireland); V. Tonini (St. Orsola Hospital–University of Bologna, Italy); M. Migliore (Polyclinic Hospital University of Catania, Italy); L. Bonavina (University of Milano, Italy); M. Valmasoni (Padova University Hospital, Italy); R. Bardini (Padova University Hospital, Italy); J. Weindelmayer (Verona Borgo Trento Hospital, Italy); M. Terashima (Shizioka Cancer Centre, Japan); R.E. White (Tenwek Hospital, Kenya); E. Alghunaim (Chest Diseases Hospital, Kuwait); M. Elhadi (Tripoli, Libya); A.M. Leon-Takahashi (National Cancer Institute, Mexico); H. Medina-Franco (National Institute of Medical Science and Nutrition Salvador Zubirán, Mexico); P.C. Lau (University Malaya Medical Centre, Malaysia); K.E. Okonta (Carez Hospital & University of Port-Harcourt Teaching Hospital, Nigeria); J. Heisterkamp (Elisabeth-TweeSteden Ziekenhuis Hospital, The Netherlands); C. Rosman (Radboudumc, The Netherlands); R. van Hillegersberg (UMC Utrecht, The Netherlands); G. Beban (Auckland City Hospital, New Zealand); R. Babor (Middlemore Hospital, New Zealand); A. Gordon (Palmerston North Hospital, New Zealand); J.I. Rossaak (Tauranga Hospital, Bay of Plenty District Health Board, New Zealand); K.M.I. Pal (Aga Khan University Hospital, Pakistan); A.U. Qureshi (Services Institute of Medical Sciences, Pakistan); S.A. Naqi (Mayo Hospital, Pakistan); A.A. Syed (Shaukat Khanum Memorial Cancer Hospital & Research Centre Lahore, Pakistan); J. Barbosa (Centro Hospitalar São João, Portugal); C.S. Vicente (Centro Hospitalar Lisboa Central, Portugal); J. Leite (Coimbra University Hospital, Portugal); J. Freire (Hospital Santa Maria, Portugal); R. Casaca (Instituto Português de Oncologia de Lisboa, Portugal); R.C.T. Costa (Instituto Português de Oncologia do Porto, Portugal); R.R. Scurtu (University Emergency Cluj County Hospital, Romania); S.S. Mogoanta (Emergency County Hospital of Craiova, Romania); C. Bolca (Marius Nasta' National Institute of Pneumology, Romania); S. Constantinoiu (St. Mary Clinical Hospital, Romania); D. Sekhniaidze (Tyumen Regional Hospital, Russia); M. Bjelović (Department for Minimally Invasive Upper Digestive Surgery, University Hospital for Digestive Surgery, Clinical Centre of Serbia, Serbia); J.B.Y. So (National University Hospital, Singapore); G. Gačevski (University Hospital Maribor, Slovenia); C. Loureiro (University Hospital of Basurto (Bilbao), Spain); M. Pera (Hospital Universitario del Mar, Spain); A. Bianchi (Palma de Mallorca, Spain); M. Moreno Gijón (Hospital Universitario Central de Asturias, Spain); J. Martín Fernández (Hospital General Universitario De Ciudad Real, Spain); M.S. Trugeda Carrera (Hospital Universitario Marqués de Valdecilla, Spain); M. Vallve-Bernal (Hospital Universitario Nuestra Señora de Candelaria, Spain); M.A. Cítores Pascual (Hospital Universitario Río Hortega de Valladolid, Spain); S. Elmahi (Shaab Teaching Hospital, Sudan), I. Halldestam (University Hospital Linköping, Sweden); J. Hedberg (Uppsala University Hospital, Sweden); S. Mönig (Geneva University Hospital, Switzerland); S. Gutknecht (Triemli Hospital Zurich, Switzerland); M. Tez (Ankara Numune Hospital, Turkey); A. Guner (Karadeniz Technical University, Turkey); T.B. Tirnaksiz (Hacettepe University Hospital, Turkey); E. Colak (University of Health Sciences, Samsun Training and Research Hospital, Turkey); B. Sevinç (Usak University Training and Research Hospital, Turkey); A. Hindmarsh (Addenbrooke’s Hospital, UK); I. Khan (Aintree University Hospital, UK); D. Khoo (Barking Havering and Redbridge NHS Trust, UK); R. Byrom (Royal Bournemouth Hospital, UK); J. Gokhale (Bradford Royal Infirmary, UK); P. Wilkerson (University Hospitals Bristol NHS Foundation Trust, UK); P. Jain (Castle Hill Hospital, UK); D. Chan (University Hospital of Coventry, UK); K. Robertson (University Hospital Crosshouse, UK); S. Iftikhar (Royal Derby Hospital, UK); R. Skipworth (Edinburgh Royal Infirmary, UK); M. Forshaw (Glasgow Royal Infirmary, UK); S. Higgs (Gloucester Royal Hospital, UK); J. Gossage (Guy’s and St Thomas’ Hospitals, UK); R. Nijjar (Heartlands Hospital, UK); Y.K.S. Viswanath (James Cook University Hospital, UK); P. Turner (Lancashire Teaching Hospitals NHS Foundation Trust, UK); S. Dexter (Leeds Teaching Hospitals NHS Trust, UK); A. Boddy (University Hospitals of Leicester NHS Trust, UK); W.H. Allum (Royal Marsden Hospital, UK); S. Oglesby (Ninewells Hospital, UK); E. Cheong (Norfolk and Norwich University Hospital, UK); D. Beardsmore (University Hospital of North Midlands, UK); R. Vohra (Nottingham University Hospital, UK); N. Maynard (Oxford University Hospitals, UK); R. Berrisford (Plymouth Hospitals NHS Trust, UK); S. Mercer (Queen Alexandra Hospital, UK); S. Puig (Queen Elizabeth Hospital Birmingham, UK); R. Melhado (Salford Royal Foundation Trust, UK); C. Kelty (Sheffield Teaching Hospitals NHS Foundation Trust, UK); T. Underwood (University Hospital Southampton NHS Foundation Trust, UK); K. Dawas (University College Hospital, UK); W. Lewis (University Hospital of Wales, UK); A. Al-Bahrani (Watford General Hospital); G. Bryce (University Hospital Wishaw, UK); M. Thomas (Mayo Clinic in Florida, USA); A.T. Arndt (Rush University Medical Centre, USA); F. Palazzo (Thomas Jefferson University, USA); R.A. Meguid (University of Colorado Hospital, USA). J. Fergusson, E. Beenen, C. Mosse, J. Salim (The Canberra Hospital, Australia); S. Cheah, T. Wright, M.P. Cerdeira, P. McQuillan (John Hunter Hospital, Australia); M. Richardson, H. Liem (Nepean Hospital, Australia); J. Spillane, M. Yacob, F. Albadawi, T. Thorpe, A. Dingle, C. Cabalag (Peter MacCallum Cancer Centre, Australia); K. Loi, O.M. Fisher (St George Public and Private Hospitals, Australia); S. Ward, M. Read, M. Johnson (St Vincent’s Hospital Melbourne, Australia); R. Bassari, H. Bui (Western Hospital, Australia); I. Cecconello, R.A.A. Sallum, J.R.M. da Rocha (Hospital das Clinicas, University of Sao Paulo School of Medicine, Brazil); L.R. Lopes, V. Tercioti Jr, J.D.S. Coelho, J.A.P. Ferrer (Unicamp University Hospital, Brazil); G. Buduhan, L. Tan, S. Srinathan (Health Sciences Centre (Winnipeg), Canada); P. Shea (Saint John Regional Hospital, Canada); J. Yeung, F. Allison, P. Carroll (Toronto General Hospital, University Health Network, Canada); F. Vargas-Barato, F. Gonzalez, J. Ortega, L. Nino-Torres, T.C. Beltrán-García (Hospital Universitario Mayor Mederi-Universidad del Rosario, Colombia); L. Castilla, M. Pineda (Hospital San Ignacio-Universidad Javeriana, Colombia); A. Bastidas, J. Gómez-Mayorga, N. Cortés, C. Cetares, S. Caceres, S. Duarte (University Hospital Fundacion Santafe de Bogota, Colombia); A. Pazdro, M. Snajdauf, H. Faltova, M. Sevcikova (Motol University Hospital, Prague, Czech Republic); P.B. Mortensen (Aalborg University Hospital, Denmark); N. Katballe, T. Ingemann, B. Morten, I. Kruhlikava (Aarhus University Hospital, Denmark); A.P. Ainswort, N.M. Stilling, J. Eckardt (Odense University Hospital, Denmark); J. Holm, M. Thorsteinsson, M. Siemsen, B. Brandt (Copenhagen University Hospital Rigshospitalet, Denmark); B. Nega, E. Teferra, A. Tizazu (Tikur Anbessa Specialized Hospital, Ethiopa); J.S. Kauppila, V. Koivukangas, S. Meriläinen (Oulu University Hospital, Finland); R. Gruetzmann, C. Krautz, G. Weber, H. Golcher (University Clinic of Erlangen, Germany); G. Emons, A. Azizian, M. Ebeling (Medical University Goettingen, Germany); S. Niebisch, N. Kreuser, G. Albanese, J. Hesse (Universitätklinium Leipzig, Germany); L. Volovnik, U. Boecher (Klinikum Rechts der Isar der TU München, Germany); M. Reeh (University Hospital Hamburg Eppendorf, Germany); S. Triantafyllou (Hippokration General Hospital University of Athens, Greece); D. Schizas, A. Michalinos, E. Baili, M. Mpoura, A. Charalabopoulos (Laikon General Hospital, Greece); D.K. Manatakis, D. Balalis (St. Savvas Cancer Hospital, Greece); J. Bolger, C. Baban, A. Mastrosimone (Beaumont Hospital, Ireland); O. McAnena, A. Quinn (University Hospital Galway, Ireland); C.B. Ó Súilleabháin, M.M. Hennessy, I. Ivanovski, H. Khizer (Mercy University Hospital, Ireland); N. Ravi, N. Donlon (St James’s Hospital, Ireland); M. Cervellera, S. Vaccari, S. Bianchini, L. Sartarelli (St. Orsola Hospital-University of Bologna, Italy); E. Asti, D. Bernardi (University of Milano, IRCCS Policlinico San Donato, Italy); S. Merigliano, L. Provenzano (Padova University Hospital—Clinica Chirurgica, Italy); M. Scarpa, L. Saadeh, B. Salmaso (Padova University Hospital-General Surgery Department, Italy); G. De Manzoni, S. Giacopuzzi, R. La Mendola, C.A. De Pasqual (Verona Borgo Trento Hospital, Italy); Y. Tsubosa, M. Niihara, T. Irino, R. Makuuchi, K. Ishii (Shizioka Cancer Centre, Japan); M. Mwachiro, A. Fekadu, A. Odera, E. Mwachiro (Tenwek Hospital, Kenya); D. AlShehab (Chest Diseases Hospital, Kuwait); H.A. Ahmed, A.O. Shebani, A. Elhadi, F.A. Elnagar, H.F. Elnagar (Tripoli, Libya); S.T. Makkai-Popa (Centre Hospitalier de Luxembourg, Luxembourg); L.F. Wong (University Malaya Medical Centre, Malaysia); T. Yunrong, S. Thanninalai, H.C. Aik, P.W. Soon, T.J. Huei (Hospital Sultanah Aminah, Malaysia); H.N.L. Basave (National Cancer Institute, Mexico); R. Cortés-González (Instituto Nacional de Ciencias Médicas y Nutrición ‘Salvador Zubirán’, Mexico); S.M. Lagarde, J.J.B. van Lanschot, C. Cords (Erasmus University Medical Centre, The Netherlands); W.A. Jansen, I. Martijnse, R. Matthijsen (Elisabeth-TweeSteden Ziekenhuis Hospital, The Netherlands); S. Bouwense, B. Klarenbeek, M. Verstegen, F. van Workum (Radboudumc, The Netherlands); J.P. Ruurda, A. van der Veen, J.W. van den Berg (UMC Utrecht, The Netherlands); N. Evenett, P. Johnston, R. Patel (Auckland City Hospital, New Zealand); A. MacCormick (Middlemore Hospital, New Zealand); M. Young (Palmerston North Hospital, New Zealand); B. Smith (Tauranga Hospital, Bay of Plenty District Health Board, New Zealand); C. Ekwunife (Carez Hospital & University of Port-Harcourt Teaching Hospital, Nigeria); A.H. Memon, K. Shaikh, A. Wajid (Aga Khan University Hospital, Pakistan); N. Khalil, M. Haris, Z.U. Mirza, S.B.A. Qudus (Services Institute of Medical Sciences, Pakistan); M.Z. Sarwar, A. Shehzadi, A. Raza, M.H. Jhanzaib (Mayo Hospital, Pakistan); J. Farmanali, Z. Zakir (Patel Hospital, Pakistan); O. Shakeel, I. Nasir, S. Khattak, M. Baig, M.A. Noor, H.H. Ahmed, A. Naeem (Shaukat Khanum Memorial Cancer Hospital & Research Centre Lahore, Pakistan); A.C. Pinho, R. da Silva (Centro Hospitalar Lisboa Central, Portugal), A. Bernardes, J.C. Campos (Coimbra University Hospital, Portugal); H. Matos, T. Braga (Hospital Santa Maria, Portugal); C. Monteiro, P. Ramos, F. Cabral (Instituto Português de Oncologia de Lisboa, Portugal); M.P. Gomes, P.C. Martins, A.M. Correia, J.F. Videira (Instituto Português de Oncologia do Porto, Portugal); C. Ciuce, R. Drasovean, R. Apostu, C. Ciuce (University Emergency Cluj County Hospital, Romania); S. Paitici, A.E. Racu, C.V. Obleaga (Emergency County Hospital of Craiova, Romania); M. Beuran, B. Stoica, C. Ciubotaru, V. Negoita (Emergency Hospital of Bucharest, Romania); I. Cordos (Marius Nasta' National Institute of Pneumology, Romania); R.D. Birla, D. Predescu, P.A. Hoara, R. Tomsa (St. Mary Clinical Hospital, Romania); V. Shneider, M. Agasiev, I. Ganjara (Tyumen Regional Hospital, Russia); D. Gunjić, M. Veselinović, T. Babič (Department for Minimally Invasive Upper Digestive Surgery, University Hospital for Digestive Surgery, Clinical Centre of Serbia, Serbia); T.S. Chin, A. Shabbir, G. Kim (National University Hospital, Singapore); A. Crnjac, H. Samo (University Hospital Maribor, Slovenia); I. Díez del Val, S. Leturio (University Hospital of Basurto (Bilbao), Spain); I. Díez del Val, S. Leturio, J.M. Ramón, M. Dal Cero, S. Rifá, M. Rico (Hospital Universitario del Mar, Spain); A. Pagan Pomar, J.A. Martinez Corcoles (Palma de Mallorca, Spain); J.L. Rodicio Miravalles, S.A. Pais, S.A. Turienzo, L.S. Alvarez (Hospital Universitario Central de Asturias, Spain); P.V. Campos, A.G. Rendo, S.S. García, E.P.G. Santos (Hospital General Universitario De Ciudad Real, Spain); E.T. Martínez, M.J. Fernández Díaz, C. Magadán Álvarez (Hospital Universitario Marqués de Valdecilla, Spain); V. Concepción Martín, C. Díaz López, A. Rosat Rodrigo, L.E. Pérez Sánchez (Hospital Universitario Nuestra Señora de Candelaria, Spain); M. Bailón Cuadrado, C. Tinoco Carrasco, E. Choolani Bhojwani, D.P. Sánchez (Hospital Universitario Río Hortega de Valladolid, Spain); M.E. Ahmed (Shaab Teaching Hospital, Sudan); T. Dzhendov (University Hospital Linköping, Sweden); F. Lindberg, M. Rutegård (Umeå University Hospital, Sweden); M. Sundbom (Uppsala University Hospital, Sweden); C. Mickael, N. Colucci (Geneva University Hospital, Switzerland); A. Schnider (Triemli Hospital Zurich, Switzerland); S. Er (Ankara Numune Hospital, Turkey); E. Kurnaz (Erzincan University Hospital, Turkey); S. Turkyilmaz, A. Turkyilmaz, R. Yildirim, B.E. Baki (Karadeniz Technical University, Turkey); N. Akkapulu (Hacettepe University Hospital, Turkey); O. Karahan, N. Damburaci (Usak University Training and Research Hospital, Turkey); R. Hardwick, P. Safranek, V. Sujendran, J. Bennett, Z. Afzal (Addenbrooke’s Hospital, UK); M. Shrotri, B. Chan, K. Exarchou, T. Gilbert (Aintree University Hospital, UK); T. Amalesh, D. Mukherjee, S. Mukherjee, T.H. Wiggins (Barking Havering and Redbridge NHS Trust, UK); R. Kennedy, S. McCain, A. Harris, G. Dobson (Belfast City Hospital, UK); N. Davies, I. Wilson, D. Mayo, D. Bennett (Royal Bournemouth Hospital, UK); R. Young, P. Manby (Bradford Royal Infirmary, UK); N. Blencowe, M. Schiller, B. Byrne (University Hospitals Bristol NHS Foundation Trust, UK); D. Mitton, V. Wong, A. Elshaer, M. Cowen (Castle Hill Hospital, UK); V. Menon, L.C. Tan, E. McLaughlin, R. Koshy (University Hospital of Coventry, UK); C. Sharp (University Hospital Crosshouse, UK); H. Brewer, N. Das, M. Cox, W. Al Khyatt, D. Worku (Royal Derby Hospital, UK); R. Iqbal, L. Walls, R. McGregor (Edinburgh Royal Infirmary, UK); G. Fullarton, A. Macdonald, C. MacKay, C. Craig (Glasgow Royal Infirmary, UK); S. Dwerryhouse, S. Hornby, S. Jaunoo, M. Wadley (Gloucester Royal Hospital, UK); C. Baker, M. Saad, M. Kelly, A. Davies, F. Di Maggio (Guy’s and St Thomas’ Hospitals, UK); S. McKay, P. Mistry, R. Singhal, O. Tucker, S. Kapoulas, S. Powell-Brett (Heartlands Hospital, UK); P. Davis, G. Bromley, L. Watson (James Cook University Hospital, UK); R. Verma, J. Ward, V. Shetty, C. Ball, K. Pursnani (Lancashire Teaching Hospitals NHS Foundation Trust, UK); A. Sarela, H. Sue Ling, S. Mehta, J. Hayden, N. To (Leeds Teaching Hospitals NHS Trust, UK); T. Palser, D. Hunter, K. Supramaniam, Z. Butt, A. Ahmed (University Hospitals of Leicester NHS Trust, UK); S. Kumar, A. Chaudry, O. Moussa (Royal Marsden Hospital, UK); A. Kordzadeh, B. Lorenzi (Mid and South Essex NHS Foundation Trust, UK). M. Wilson, P. Patil, I. Noaman (Ninewells Hospital, UK); J. Willem (Norfolk and Norwich University Hospital); G. Bouras, R. Evans, M. Singh, H. Warrilow, A. Ahmad (University Hospital of North Midlands, UK); N. Tewari, F. Yanni, J. Couch, E. Theophilidou, J.J. Reilly, P. Singh (Nottingham University Hospital, UK); G. van Boxel, K. Akbari, D. Zanotti, B. Sgromo (Oxford University Hospitals, UK); G. Sanders, T. Wheatley, A. Ariyarathenam, A. Reece-Smith, L. Humphreys (Plymouth Hospitals NHS Trust, UK); C. Choh, N. Carter, B. Knight, P. Pucher (Queen Alexandra Hospital, UK); A. Athanasiou, I. Mohamed, B. Tan, M. Abdulrahman (Queen Elizabeth Hospital Birmingham, UK); J. Vickers, K. Akhtar, R. Chaparala, R. Brown, M.M.A. Alasmar (Salford Royal Foundation Trust, UK); R. Ackroyd, K. Patel, A. Tamhankar, A. Wyman (Sheffield Teaching Hospitals NHS Foundation Trust, UK); R. Walker, B. Grace (University Hospital Southampton NHS Foundation Trust, UK); N. Abbassi, N. Slim, L. Ioannidi (University College Hospital, UK); G. Blackshaw, T. Havard, X. Escofet, A. Powell (University Hospital of Wales, UK); A. Owera, F. Rashid, P. Jambulingam, J. Padickakudi (Watford General Hospital, UK); H. Ben-Younes, K. McCormack (University Hospital Wishaw, UK); I.A. Makey (Mayo Clinic in Florida, USA); M.K. Karush, C.W. Seder, M.J. Liptay, G. Chmielewski (Rush University Medical Centre, USA); E.L. Rosato, A.C. Berger, R. Zheng, E. Okolo (Thomas Jefferson University, USA); A. Singh, C.D. Scott, M.J. Weyant, J.D. Mitchell (University of Colorado Hospital, USA).

## Supplementary Material

zrab132_Supplementary_Data
