# Supplementary material for: Postoperative outcomes in oesophagectomy with trainee involvement
Source: BJS Open. 2022 Jan 17;5(6):zrab132. doi: 10.1093/bjsopen/zrab132 (PMC8763367; doi:10.1093/bjsopen/zrab132)
Supplement: zrab132_Supplementary_Data [file zrab132_supplementary_data.zip › Supplementary_Appendix_1.docx]

**Appendix S1**

**OGAA Author List**

**Writing Committee:** Evans RPT, Kamarajah SK, Bundred J, Nepogodiev D, Hodson J, van Hillegersberg R, Gossage J, Vohra R, Griffiths EA, Singh P,

**Data Analysis**: Evans RPT, Hodson J, Kamarajah SK, Griffiths EA, Singh P

**Steering Committee**: Alderson D, Bundred J, Evans RPT, Gossage J, Griffiths EA, Jefferies B, Kamarajah SK, McKay S, Mohamed I, Nepogodiev D, Siaw- Acheampong K, Singh P, van Hillegersberg R, Vohra R, Wanigasooriya K, Whitehouse T.

**National Leads:** Gjata A (Albania), Moreno JI (Argentina), Takeda FR (Brazil), Kidane B (Canada), Guevara Castro R (Colombia), Harustiak T (Czech Republic), Bekele A (Ethiopia), Kechagias A (Finland), Gockel I (Germany), Kennedy A (Ireland), Da Roit A (Italy), Bagajevas A (Lithuania), Azagra JS (Luxembourg), Mahendran HA (Malaysia), Mejía-Fernández L (Mexico), Wijnhoven BPL (Netherlands), El Kafsi J (New Zealand), Sayyed RH (Pakistan), Sousa M (Portugal), Sampaio AS (Portugal), Negoi I (Romania), Blanco R (Spain), Wallner B (Sweden), Schneider PM (Switzerland), Hsu PK (Taiwan), Isik A (Turkey)

**Site Leads:**

Gananadha S (The Canberra Hospital, Australia); Wills V (John Hunter Hospital, Australia); Devadas M (Nepean Hospital, Australia); Duong C (Peter MacCallum Cancer Centre, Australia); Talbot M (St George Public and Private Hospitals, Australia); Hii MW (St Vincent's Hospital Melbourne, Australia); Jacobs R (Western Hospital, Victoria, Australia); Andreollo NA (Unicamp University Hospital, Brazil); Johnston B (Saint John Regional Hospital, Canada); Darling G (Toronto General Hospital, University Health Network, Canada); Isaza-Restrepo A (Hospital Universitario Mayor Mederi-Universidad del Rosario, Colombia); Rosero G (Hospital San Ignacio-Universidad Javeriana, Colombia); Arias- Amézquita F (University Hospital Fundacion Santafe de Bogota, Colombia); Raptis D (University Clinic of Erlangen, Germany); Gaedcke J (Medical Unversity Goettingen, Germany); Reim D (Klinikum Rechts der Isar der TU München, Germany); Izbicki J (University Hospital Hamburg Eppendorf, Germany); Egberts JH(University Hospital Kiel, Germany); Dikinis S (Aalborg University Hospital, Denmark); Kjaer DW (Aarhus University Hospital, Denmark); Larsen MH (Odense University Hospital, Denmark); Achiam MP (Copenhagen University hospital Rigshospitalet, Denmark); Saarnio J (Oulu University Hospital, Finland); Theodorou D (Hippokration General Hospital University of Athens, Greece); Liakakos T (Laikon General Hospital, Greece); Korkolis DP (St. Savvas Cancer Hospital, Greece); Robb WB (Beaumont Hospital, Ireland); Collins C (University Hospital Galway, Ireland); Murphy T (Mercy University Hospital, Ireland); Reynolds J (St James's Hospital, Dublin, Ireland); Tonini V (St. Orsola Hospital- University of Bologna, Italy); Migliore M (Polyclinic Hospital University of Catania, Italy); Bonavina L (University of Milano, IRCCS Policlinico San Donato, Department of General and Foregut Surgery, Italy); Valmasoni M (Padova University Hospital - Clinica Chirurgica 3, Italy); Bardini R (Padova University Hospital- General Surgery Department, Italy); Weindelmayer J (Verona Borgo Trento Hospital, Italy); Terashima M (Shizioka Cancer Centre, Japan); White RE (Tenwek Hospital, Kenya); Alghunaim E (Chest Diseases Hospital, Kuwait); Elhadi M (Tripoli, Libya); Leon-Takahashi AM (National Cancer Institute, Mexico); Medina-Franco H (National Institute of Medical Science and Nutrition Salvador Zubirán, Mexico); Lau PC (University Malaya Medical Centre, Malaysia); Okonta KE (Carez Hospital & University of Port-Harcourt Teaching Hospital, Nigeria); Heisterkamp J (Elisabeth-TweeSteden Ziekenhuis Hospital, Netherlands); Rosman C (Radboudumc, Netherlands); van Hillegersberg R (UMC Utrecht, Netherlands); Beban G (Auckland City Hospital, New Zealand); Babor R (Middlemore Hospital, New Zealand); Gordon A (Palmerston North Hospital, New Zealand); Rossaak JI (Tauranga Hospital, Bay of Plenty District Health Board, New Zealand); Pal KMI (Aga Khan University Hospital, Pakistan); Qureshi AU (Services Institute of Medical Sciences, Lahore, Pakistan); Naqi SA (Mayo Hospital, Lahore, Pakistan); Syed AA (Shaukat Khanum Memorial Cancer Hospital & Research Centre Lahore, Pakistan); Barbosa J (Centro Hospitalar São João, Portugal); Vicente CS (Centro Hospitalar Lisboa Central, Portugal); Leite J (Coimbra University Hospital, Portugal); Freire J (Hospital Santa Maria, Portugal); Casaca R (Instituto Português de Oncologia de Lisboa, Portugal); Costa RCT (Instituto Português de Oncologia do Porto, Portugal); Scurtu RR (University Emergency Cluj County Hospital, Romania); Mogoanta SS (Emergency County Hospital of Craiova, Romania); Bolca C (Marius Nasta' National Institute of Pneumology, Romania); Constantinoiu S (St. Mary Clinical Hospital, Romania); Sekhniaidze D (Tyumen Regional Hospital, Russia); Bjelović M (Department for Minimally Invasive Upper Digestive Surgery, University Hospital for Digestive Surgery, Clinical Centre of Serbia, Belgrade, Serbia); So JBY (National University Hospital, Singapore); Gačevski G (University Hospital Maribor, Slovenia); Loureiro C (University Hospital of Basurto (Bilbao), Spain); Pera M (Hospital Universitario del Mar, Spain); Bianchi A (Palma de Mallorca, Spain); Moreno Gijón M (Hospital Universitario Central de Asturias, Spain); Martín Fernández J (Hospital General Universitario De Ciudad Real, Spain); Trugeda Carrera MS (Hospital Universitario Marqués de Valdecilla, Spain); Vallve-Bernal M (Hospital Universitario Nuestra Señora de Candelaria, Spain); Cítores Pascual MA (Hospital Universitario Río Hortega de Valladolid, Spain); Elmahi S (Shaab Teaching Hospital, Sudan), Halldestam I (University Hospital Linköping, Sweden); Hedberg J (Uppsala University Hospital, Sweden); Mönig S (Geneva University Hospital, Switzerland); Gutknecht S (Triemli Hospital Zurich, Switzerland); Tez M (Ankara Numune Hospital, Turkey); Guner A (Karadeniz Technical University, Turkey); Tirnaksiz MB (Hacettepe University Hospital, Turkey); Colak E (University of Health Sciences, Samsun Training and Research Hospital, Turkey); Sevinç B (Usak University Training and Research Hospital, Turkey); Hindmarsh A (Addenbrooke's Hospital, Cambridge, United Kingdom (UK)); Khan I (Aintree University Hospital, Liverpool, UK); Khoo D (Barking Havering and Redbridge NHS Trust, UK); Byrom R (Royal Bournemouth Hospital, UK); Gokhale J (Bradford Royal Infirmary, UK); Wilkerson P (University Hospitals Bristol NHS Foundation Trust, UK); Jain P (Castle Hill Hospital, UK); Chan D (University Hospital of Coventry, UK); Robertson K (University Hospital Crosshouse, UK); Iftikhar S (Royal Derby Hospital, UK); Skipworth R (Edinburgh Royal Infirmary, UK); Forshaw M (Glasgow Royal Infirmary, UK); Higgs S (Gloucester Royal Hospital, UK); Gossage J (Guy's and St Thomas's Hospitals, UK); Nijjar R (Heartlands Hospital, UK); Viswanath YKS (James Cook University Hospital, UK); Turner P (Lancashire Teaching Hospitals NHS Foundation Trust, UK); Dexter S (Leeds Teaching Hospitals NHS Trust, UK); Boddy A (University Hospitals of Leicester NHS Trust, UK); Allum WH (Royal Marsden Hospital, UK); Oglesby S (Ninewells Hospital, UK); Cheong E (Norfolk and Norwich University Hospital, UK); Beardsmore D (University Hospital of North Midlands, UK); Vohra R (Nottingham University Hospital, UK); Maynard N (Oxford University Hospitals, UK); Berrisford R (Plymouth Hospitals NHS Trust, UK); Mercer S (Queen Alexandra Hospital, Portsmouth, UK); Puig S (Queen Elizabeth Hospital Birmingham, UK); Melhado R (Salford Royal Foundation Trust, UK); Kelty C (Sheffield Teaching Hospitals NHS Foundation Trust, UK); Underwood T (University Hospital Southampton NHS Foundation Trust, UK); Dawas K (University College Hospital, UK); Lewis W (University Hospital of Wales, UK); Al-Bahrani A (Watford General Hospital); Bryce G (University Hospital Wishaw, UK); Thomas M (Mayo Clinic in Florida, United States of America (USA)); Arndt AT (Rush University Medical Centre, USA); Palazzo F (Thomas Jefferson University, USA); Meguid RA (University of Colorado Hospital, USA)

**Collaborators:**

Fergusson J, Beenen E, Mosse C, Salim J (The Canberra Hospital, Australia); Cheah S, Wright T, Cerdeira MP, McQuillan P (John Hunter Hospital, Australia); Richardson M, Liem H ( Nepean Hospital, Australia); Spillane J, Yacob M, Albadawi F, Thorpe T, Dingle A, Cabalag C (Peter MacCallum Cancer Centre, Australia); Loi K, Fisher OM (St George Public and Private Hospitals, Australia); Ward S, Read M, Johnson M (St Vincent's Hospital Melbourne, Australia); Bassari R, Bui H (Western Hospital, Victoria); Cecconello I, Sallum RAA, da Rocha JRM (Hospital das Clinicas, University of Sao Paulo School of Medicine, Brazil); Lopes LR, Tercioti V Jr, Coelho JDS, Ferrer JAP (Unicamp University Hospital, Brazil); Buduhan G, Tan L, Srinathan S (Health Sciences Centre (Winnipeg)); Shea P (Saint John Regional Hospital, Canada); Yeung J, Allison F, Carroll P (Toronto General Hospital, University Health Network, Canada); Vargas-Barato F, Gonzalez F, Ortega J, Nino-Torres L, Beltrán-García TC (Hospital Universitario Mayor Mederi-Universidad del Rosario, Colombia); Castilla L, Pineda M (Hospital San Ignacio-Universidad Javeriana, Colombia); Bastidas A, Gómez-Mayorga J, Cortés N, Cetares C, Caceres S, Duarte S (University Hospital Fundacion Santafe de Bogota, Colombia); Pazdro A, Snajdauf M, Faltova H, Sevcikova M (Motol University Hospital, Prague, Czech Republic); Mortensen PB (Aalborg University Hospital, Denmark); Katballe N, Ingemann T, Morten B, Kruhlikava I (Aarhus University Hospital, Denmark); Ainswort AP, Stilling NM, Eckardt J (Odense University Hospital, Denmark); Holm J, Thorsteinsson M, Siemsen M, Brandt B (Copenhagen University hospital Rigshospitalet, Denmark); Nega B, Teferra E, Tizazu A (Tikur Anbessa Specialized Hospital, Ethiopa); Kauppila JS, Koivukangas V, Meriläinen S (Oulu University Hospital, Finland); Gruetzmann R, Krautz C, Weber G, Golcher H (University Clinic of Erlangen, Germany); Emons G, Azizian A, Ebeling M (Medical University Goettingen, Germany); Niebisch S, Kreuser N, Albanese G, Hesse J (Universitätklinium Leipzig, Germany); Volovnik L, Boecher U (Klinikum Rechts der Isar der TU München, Germany); Reeh M (University Hospital Hamburg Eppendorf, Germany); Triantafyllou S (Hippokration General Hospital University of Athens, Greece); Schizas D, Michalinos A, Baili E, Mpoura M, Charalabopoulos A (Laikon General Hospital, Greece); Manatakis DK, Balalis D (St. Savvas Cancer Hospital, Greece); Bolger J, Baban C, Mastrosimone A (Beaumont Hospital, Ireland); McAnena O, Quinn A (University Hospital Galway, Ireland); Ó Súilleabháin CB, Hennessy MM, Ivanovski I, Khizer H (Mercy University Hospital, Ireland); Ravi N, Donlon N (St James's Hospital, Dublin, Ireland); Cervellera M, Vaccari S, Bianchini S, Sartarelli l (St. Orsola Hospital- University of Bologna, Italy); Asti E, Bernardi D (University of Milano, IRCCS Policlinico San Donato, Department of General and Foregut Surgery, Italy); Merigliano S, Provenzano L (Padova University Hospital - Clinica Chirurgica, Italy); Scarpa M, Saadeh L, Salmaso B (Padova University Hospital- General Surgery Department, Italy); De Manzoni G, Giacopuzzi S, La Mendola R, De Pasqual CA (Verona Borgo Trento Hospital, Italy); Tsubosa Y, Niihara M, Irino T, Makuuchi R, Ishii K (Shizioka Cancer Centre, Japan); Mwachiro M, Fekadu A, Odera A, Mwachiro E (Tenwek Hospital, Kenya); AlShehab D (Chest diseases hospital, Kuwait); Ahmed HA, Shebani AO, Elhadi A, Elnagar FA, Elnagar HF (Tripoli, Libya); Makkai-Popa ST (Centre Hospitalier de Luxembourg, Luxembourg); Wong LF (University Malaya Medical Centre, Malaysia); Yunrong T, Thanninalai S, Aik HC, Soon PW, Huei TJ (Hospital Sultanah Aminah, Malaysia); Basave HNL ( National Cancer Institute, Mexico); Cortés-González R (Instituto Nacional de Ciencias Médicas y Nutrición 'Salvador Zubirán', Mexico); Lagarde SM, van Lanschot JJB, Cords C (Erasmus University Medical Centre, Rotterdam, Netherlands); Jansen WA, Martijnse I, Matthijsen R (Elisabeth-TweeSteden Ziekenhuis Hospital, Netherlands); Bouwense S, Klarenbeek B, Verstegen M, van Workum F (Radboudumc, Netherlands); Ruurda JP, van der Veen A, van den Berg J W (UMC Utrecht, Netherlands); Evenett N, Johnston P, Patel R (Auckland City Hospital, New Zealand); MacCormick A (Middlemore Hospital, New Zealand); Young M (Palmerston North Hospital); Smith B (Tauranga Hospital, Bay of Plenty District Health Board, New Zealand); Ekwunife C (Carez Hospital & University of Port-Harcourt Teaching Hospital, Nigeria); Memon AH, Shaikh K, Wajid A (Aga Khan University Hospital, Pakistan); Khalil N, Haris M, Mirza ZU, Qudus SBA (Services Institute of Medical Sciences, Lahore, Pakistan); Sarwar MZ, Shehzadi A, Raza A, Jhanzaib MH (Mayo Hospital, Lahore, Pakistan); Farmanali J, Zakir Z (Patel Hospital, Pakistan); Shakeel O, Nasir I, Khattak S, Baig M, Noor MA, Ahmed HH, Naeem A (Shaukat Khanum Memorial Cancer Hospital & Research Centre Lahore, Pakistan); Pinho AC, da Silva R (Centro Hospitalar Lisboa Central, Portugal), Bernardes A, Campos JC (Coimbra University Hospital, Portugal); Matos H, Braga T (Hospital Santa Maria, Portugal); Monteiro C, Ramos P, Cabral F (Instituto Português de Oncologia de Lisboa, Portugal); Gomes MP, Martins PC, Correia AM, Videira JF (Instituto Português de Oncologia do Porto, Portugal); Ciuce C, Drasovean R, Apostu R, Ciuce C (University Emergency Cluj County Hospital, Romania); Paitici S, Racu AE, Obleaga CV (Emergency County Hospital of Craiova, Romania); Beuran M, Stoica B, Ciubotaru C, Negoita V (Emergency Hospital of Bucharest, Romania); Cordos I (Marius Nasta' National Institute of Pneumology, Romania); Birla RD, Predescu D, Hoara PA, Tomsa R (St. Mary Clinical Hospital, Romania); Shneider V, Agasiev M, Ganjara I (Tyumen Regional Hospital, Russia); Gunjić D, Veselinović M, Babič T (Department for Minimally Invasive Upper Digestive Surgery, University Hospital for Digestive Surgery, Clinical Centre of Serbia, Belgrade, Serbia); Chin TS, Shabbir A, Kim G (National University Hospital, Singapore); Crnjac A, Samo H (University Hospital Maribor, Slovenia); Díez del Val I, Leturio S (University Hospital of Basurto (Bilbao), Spain); Díez del Val I, Leturio S, Ramón JM, Dal Cero M, Rifá S, Rico M (Hospital Universitario del Mar, Spain); Pagan Pomar A, Martinez Corcoles JA (Palma de Mallorca, Spain); Rodicio Miravalles JL, Pais SA, Turienzo SA, Alvarez LS (Hospital Universitario Central de Asturias, Spain); Campos PV, Rendo AG, García SS, Santos EPG (Hospital General Universitario De Ciudad Real, Spain); Martínez ET, Fernández Díaz MJ, Magadán Álvarez C (Hospital Universitario Marqués de Valdecilla, Spain); Concepción Martín V, Díaz López C, Rosat Rodrigo A, Pérez Sánchez LE (Hospital Universitario Nuestra Señora de Candelaria, Spain); Bailón Cuadrado M, Tinoco Carrasco C, Choolani Bhojwani E, Sánchez DP (Hospital Universitario Río Hortega de Valladolid, Spain); Ahmed ME (Shaab Teaching Hospital, Sudan); Dzhendov T (University Hospital Linköping, Sweden); Lindberg F, Rutegård M (Umeå University Hospital, Sweden); Sundbom M (Uppsala University Hospital, Sweden); Mickael C, Colucci N (Geneva University Hospital, Switzerland); Schnider A (Triemli Hospital Zurich, Switzerland); Er S (Ankara Numune Hospital, Turkey); Kurnaz E (Erzincan University Hospital, Turkey); Turkyilmaz S, Turkyilmaz A, Yildirim R, Baki BE (Karadeniz Technical University, Turkey); Akkapulu N (Hacettepe University Hospital, Turkey); Karahan O, Damburaci N (Usak University Training and Research Hospital, Turkey); Hardwick R, Safranek P, Sujendran V, Bennett J, Afzal Z (Addenbrooke's Hospital, Cambridge, United Kingdom (UK)); Shrotri M, Chan B, Exarchou K, Gilbert T (Aintree University Hospital, Liverpool, UK); Amalesh T, Mukherjee D, Mukherjee S, Wiggins TH (Barking Havering and Redbridge NHS Trust, UK); Kennedy R, McCain S, Harris A, Dobson G (Belfast City Hospital, UK); Davies N, Wilson I, Mayo D, Bennett D (Royal Bournemouth Hospital, UK); Young R, Manby P (Bradford Royal Infirmary, UK); Blencowe N, Schiller M, Byrne B (University Hospitals Bristol NHS Foundation Trust, UK); Mitton D, Wong V, Elshaer A, Cowen M (Castle Hill Hospital, UK); Menon V, Tan LC, McLaughlin E, Koshy R (University Hospital of Coventry, UK); Sharp C (University Hospital Crosshouse, UK); Brewer H, Das N, Cox M, Al Khyatt W, Worku D (Royal Derby Hospital, UK); Iqbal R, Walls L, McGregor R (Edinburgh Royal Infirmary, UK); Fullarton G, Macdonald A, MacKay C, Craig C (Glasgow Royal Infirmary, UK); Dwerryhouse S, Hornby S, Jaunoo S, Wadley M ( Gloucester Royal Hospital, UK); Baker C, Saad M, Kelly M, Davies A, Di Maggio F (Guy's and St Thomas's Hospitals, UK); McKay S, Mistry P, Singhal R, Tucker O, Kapoulas S, Powell-Brett S (Heartlands Hospital, UK); Davis P, Bromley G, Watson L (James Cook University Hospital, UK); Verma R, Ward J, Shetty V, Ball C, Pursnani K (Lancashire Teaching Hospitals NHS Foundation Trust, UK); Sarela A, Sue Ling H, Mehta S, Hayden J, To N (Leeds Teaching Hospitals NHS Trust, UK); Palser T, Hunter D, Supramaniam K, Butt Z, Ahmed A (University Hospitals of Leicester NHS Trust, UK); Kumar S, Chaudry A, Moussa O (Royal Marsden Hospital, UK); Kordzadeh A, Lorenzi B (Mid and South Essex NHS Foundation Trust, UK)

Wilson M, Patil P, Noaman I (Ninewells Hospital, UK); Willem J (Norfolk and Norwich University Hospital); Bouras G, Evans R, Singh M, Warrilow H, Ahmad A (University Hospital of North Midlands, UK); Tewari N, Yanni F, Couch J, Theophilidou E, Reilly JJ, Singh P (Nottingham University Hospital, UK); van Boxel Gijs, Akbari K, Zanotti D, Sgromo B (Oxford University Hospitals); Sanders G, Wheatley T, Ariyarathenam A, Reece-Smith A, Humphreys L (Plymouth Hospitals NHS Trust, UK); Choh C, Carter N, Knight B, Pucher P (Queen Alexandra Hospital, Portsmouth, UK); Athanasiou A, Mohamed I, Tan B, Abdulrahman M (Queen Elizabeth Hospital Birmingham, UK); Vickers J, Akhtar K, Chaparala R, Brown R, Alasmar MMA (Salford Royal Foundation Trust, UK); Ackroyd R, Patel K, Tamhankar A, Wyman A (Sheffield Teaching Hospitals NHS Foundation Trust, UK); Walker R, Grace B (University Hospital Southampton NHS Foundation Trust, UK); Abbassi N, Slim N, Ioannidi L (University College Hospital, UK); Blackshaw G, Havard T, Escofet X, Powell A (University Hospital of Wales, UK); Owera A, Rashid F, Jambulingam P, Padickakudi J (Watford General Hospital, UK); Ben-Younes H, Mccormack K (University Hospital Wishaw, UK); Makey IA (Mayo Clinic in Florida, United States of America (USA)); Karush MK, Seder CW, Liptay MJ, Chmielewski G (Rush University Medical Centre, USA); Rosato EL, Berger AC, Zheng R, Okolo E (Thomas Jefferson University, USA); Singh A, Scott CD, Weyant MJ, Mitchell JD (University of Colorado Hospital, USA).
