# Supplementary material for: Postoperative outcomes in oesophagectomy with trainee involvement
Source: BJS Open. 2022 Jan 17;5(6):zrab132. doi: 10.1093/bjsopen/zrab132 (PMC8763367; doi:10.1093/bjsopen/zrab132)
Supplement: zrab132_Supplementary_Data [file zrab132_supplementary_data.zip › Supplementary_Tables.docx]

***Table S1. Multivariable analyses of anastomotic leak/conduit necrosis by location of anastomosis***

|  | **Chest Anastomoses** | | **Neck Anastomoses** | |
| --- | --- | --- | --- | --- |
|  | ***Odds Ratio (95% CI)*** | ***p-Value*** | ***Odds Ratio (95% CI)*** | ***p-Value*** |
| Trainee Involvement |  | **0.043** |  | 0.090 |
| *Neither* | - | - | - | - |
| *Abdomen* | 0.45 (0.25 - 0.80) | **0.006** | 0.90 (0.36 - 2.22) | 0.820 |
| *Chest/Neck* | 0.73 (0.40 - 1.32) | 0.293 | 1.46 (0.34 - 6.20) | 0.611 |
| *Abdomen+Chest/Neck* | 0.94 (0.60 - 1.48) | 0.788 | 0.47 (0.24 - 0.90) | 0.022 |
| Body Mass Index (kg/m^2^) |  | **0.046** |  | *NS* |
| *<18.5* | 1.12 (0.40 - 3.12) | 0.836 | - | - |
| *18.5-24.9* | - | - | - | - |
| *25.0-29.9* | 1.41 (1.03 - 1.94) | **0.032** | - | - |
| *30+* | 1.65 (1.12 - 2.42) | **0.011** | - | - |
| ASA Grade |  | *NS* |  | **<0.001** |
| *1* | - | - | - | - |
| *2* | - | - | 0.48 (0.24 - 0.95) | **0.035** |
| *3-4* | - | - | 1.28 (0.69 - 2.36) | 0.429 |
| Smoking Status |  | *NS* |  | 0.075 |
| *Never* | - | - | - | - |
| *Ex-Smoker* | - | - | 1.60 (0.94 - 2.73) | 0.081 |
| *Current* | - | - | 2.15 (1.11 - 4.18) | 0.024 |
| COPD (Yes) | 1.95 (1.28 - 2.96) | **0.002** | 1.55 (0.93 - 2.57) | 0.092 |
| Cardiovascular Disease (Yes) | 1.91 (1.35 - 2.69) | **<0.001** | - | *NS* |
| Histology |  | 0.056 |  | *NS* |
| *Adenocarcinoma* | - | - | - | - |
| *Squamous Cell Carcinoma* | 1.21 (0.75 - 1.95) | 0.437 | - | - |
| *Other* | 2.56 (1.17 - 5.57) | 0.018 | - | - |
| Pre-Operative Nutrition |  | *NS* |  | 0.080 |
| *None* | - | - | - | - |
| *Oral Supplements* | - | - | 0.59 (0.38 - 0.94) | 0.026 |
| *Enteral Tube Nutrition* | - | - | 0.91 (0.48 - 1.75) | 0.787 |
| *Parenteral Nutrition* | - | - | 0.26 (0.03 - 2.26) | 0.220 |
| Neoadjuvant Therapy |  | 0.084 |  | 0.059 |
| *None* | - | - | - | - |
| *Chemoradiotherapy* | 0.90 (0.61 - 1.32) | 0.580 | 1.74 (0.90 - 3.37) | 0.101 |
| *Chemotherapy Alone* | 0.66 (0.44 - 0.98) | 0.040 | 1.41 (0.68 - 2.93) | 0.355 |
| *Radiotherapy Alone* | NA* | NA* | 4.83 (1.56 - 14.96) | 0.006 |
| Abdominal Phase (Open) | 0.64 (0.45 - 0.92) | **0.014** | - | *NS* |
| Gastric Tube (Wide/Whole Stomach) | 1.44 (1.00 - 2.06) | **0.047** | - | *NS* |
| Pyloric Procedures |  | *NS* |  | **0.005** |
| *Not Performed* | - | - | - | - |
| *Botox / Dilatation* | - | - | 1.14 (0.53 - 2.47) | 0.732 |
| *Pyloromyotomy* | - | - | 3.94 (1.60 - 9.73) | **0.003** |
| *Pyloroplasty* | - | - | 0.75 (0.36 - 1.57) | 0.448 |

*Results are from multivariable generalised estimating equation models, with separate models produced for chest and neck anastomoses. In each case, the trainee involvement variable was entered at the first step, and a backwards stepwise approach was used to select other factors from* ***Table 1/2*** *for inclusion in the final parsimonious models. Only those factors selected for inclusion in one of the two models are reported in the table. The final models were based on N=1633 (214 events) for chest anastomoses, and N=486 (95 events) for neck anastomoses, after excluding cases with missing data. Performance of the multivariable models was reasonable for both chest and neck anastomoses, with areas under the ROC curve of 0.65 and 0.74, and no evidence of poor fit (Hosmer-Lemeshow test: p=0.126 and 0.411). Bold p-values are significant at p<0.05. NS=not selected for inclusion by the stepwise procedure. COPD=Chronic obstructive pulmonary disease. *Patients in the “radiotherapy alone” group were excluded from the analysis of chest anastomoses, as there were no outcomes in this group, making odds ratios incalculable.*

***Table S2. Multivariable analyses of Clavien-Dindo Grade III-V complications by location of anastomosis***

|  | **Chest Anastomoses** | | **Neck Anastomoses** | |
| --- | --- | --- | --- | --- |
|  | ***Odds Ratio (95% CI)*** | ***p-Value*** | ***Odds Ratio (95% CI)*** | ***p-Value*** |
| Trainee Involvement |  | 0.896 |  | 0.185 |
| *Neither* | - | - | - | - |
| *Abdomen* | 1.11 (0.73 - 1.70) | 0.625 | 0.58 (0.24 - 1.38) | 0.217 |
| *Chest/Neck* | 0.88 (0.48 - 1.60) | 0.668 | 0.69 (0.21 - 2.30) | 0.541 |
| *Abdomen+Chest/Neck* | 1.03 (0.63 - 1.67) | 0.919 | 0.55 (0.31 - 0.99) | 0.045 |
| Gender (Male) | - | *NS* | 1.79 (1.00 - 3.20) | **0.050** |
| Charlson Comorbidity Index (per Point)* | - | *NS* | 1.13 (1.00 - 1.28) | **0.049** |
| ASA Grade |  | 0.077 |  | *NS* |
| *1* | - | - | - | - |
| *2* | 1.16 (0.82 - 1.65) | 0.392 | - | - |
| *3-4* | 1.53 (1.05 - 2.24) | 0.027 | - | - |
| ECOG Status |  | **<0.001** |  | *NS* |
| *0* | - | - | - | - |
| *1* | 1.56 (1.23 - 1.97) | **<0.001** | - | - |
| *2* | 2.10 (1.29 - 3.41) | **0.003** | - | - |
| *3-4* | 3.15 (1.15 - 8.68) | **0.026** | NA** | - |
| Cardiovascular Disease | 1.36 (1.01 - 1.83) | **0.043** | - | *NS* |
| Histology |  | 0.069 |  | *NS* |
| *Adenocarcinoma* | - | - | - | - |
| *Squamous Cell Carcinoma* | 1.28 (0.94 - 1.73) | 0.114 | - | - |
| *Other* | 1.88 (0.93 - 3.78) | 0.077 | - | - |
| Post-Operative Nutrition |  | 0.074 |  | *NS* |
| *None* | - | - | - | - |
| *Feeding Jejunostomy* | 1.23 (0.91 - 1.65) | 0.174 | - | - |
| *Nasojejunal tube* | 0.70 (0.39 - 1.23) | 0.213 | - | - |
| Gastric Tube (Wide/Whole Stomach) | 1.30 (0.96 - 1.77) | 0.094 | - | *NS* |

*Results are from multivariable generalised estimating equation models, with separate models produced for chest and neck anastomoses. In each case, the trainee involvement variable was entered at the first step, and a backwards stepwise approach was used to select other factors from* ***Table 1/2*** *for inclusion in the final parsimonious models. Only those factors selected for inclusion in one of the two models are reported in the table. The final models were based on N=1633 (407 events) for chest anastomoses, and N=486 (138 events) for neck anastomoses, after excluding cases with missing data. Performance of the multivariable models was reasonable for both chest and neck anastomoses, with areas under the ROC curve of 0.60 and 0.62. There was no evidence of poor fit for the model of chest anastomoses (Hosmer-Lemeshow test: p=0.952); however, there was some evidence of sub-optimal fit for the model of neck anastomoses (p=0.042). Bold p-values are significant at p<0.05. NS=not selected for inclusion by the stepwise procedure. *The Charlson Comorbidity Index was treated as a continuous covariate, hence the odds ratio represents in the increased risk per one point increase in the score. **Patients with ECOG performance status of 3-4 were excluded from the analysis of neck anastomoses, as the small sample size in this group resulted in non-convergence of the model.*
