# Supplementary figures and images for: Postoperative outcomes in oesophagectomy with trainee involvement
Source: BJS Open. 2022 Jan 17;5(6):zrab132. doi: 10.1093/bjsopen/zrab132 (PMC8763367; doi:10.1093/bjsopen/zrab132)

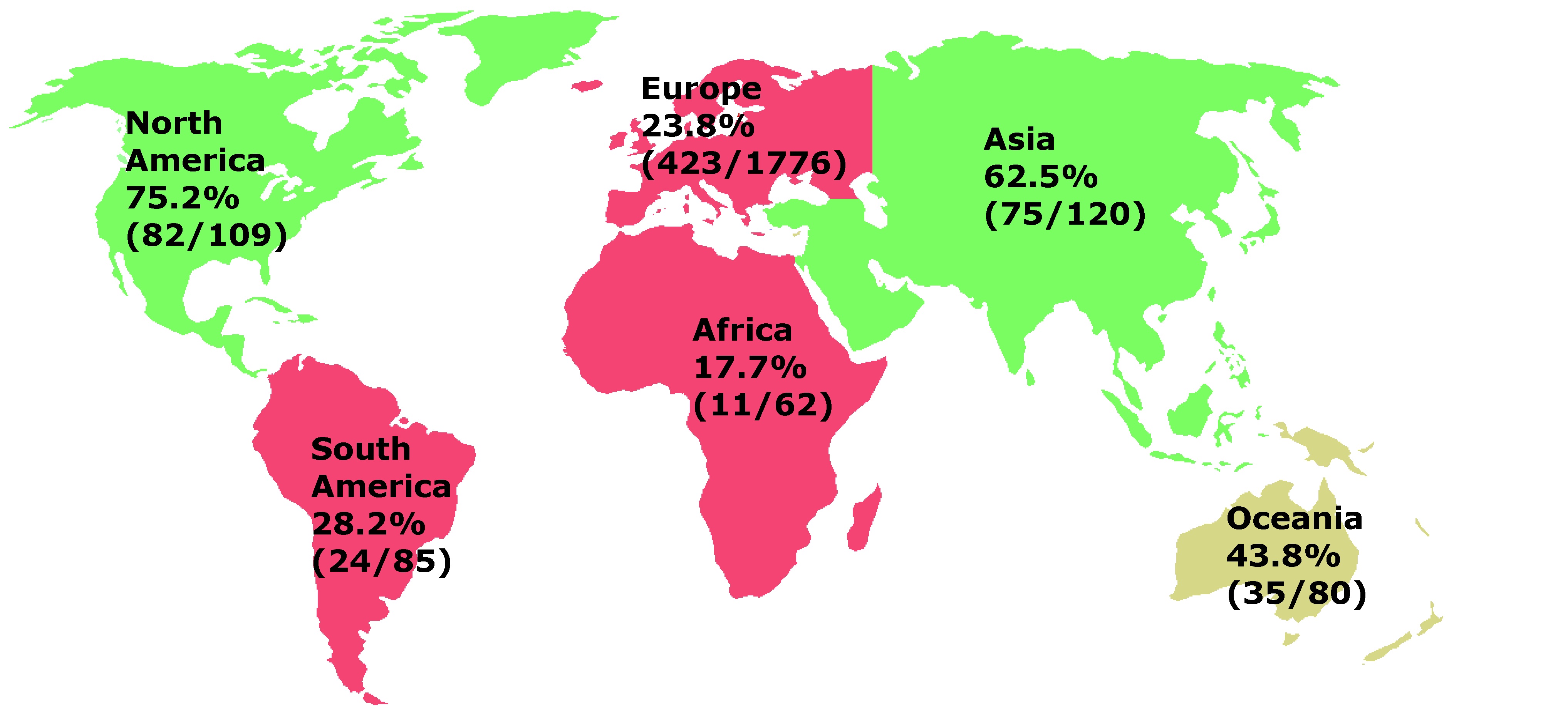

Supplement: zrab132_Supplementary_Data [file zrab132_supplementary_data.zip › Supplementary_Figure_1.jpg]
